# Application of Serum NADPH Oxidase 2 Levels for Predicting 180‐Day Clinical Outcomes Following Severe Traumatic Brain Injury: A Prospective Cohort Analysis

**DOI:** 10.1002/brb3.70692

**Published:** 2025-07-11

**Authors:** Chang Su, Dapu Shen, Junlong Xu, Miaomiao Chen, Heng He, Jianping Ye

**Affiliations:** ^1^ Department of Neurosurgery Lishui Hospital of Wenzhou Medical University, Lishui City People's Hospital Lishui Zhejiang Province China; ^2^ Department of Intensive Care Unit Lishui Hospital of Wenzhou Medical University, Lishui People's Hospital Lishui Zhejiang Province China

**Keywords:** mortality, NOX2, poor prognosis, severity, severe traumatic brain injury

## Abstract

**Objectives:**

Nicotinamide adenine dinucleotide phosphate oxidase 2 (NOX2) affects oxidative response to acute brain injury. We set out to determine if there are connections between serum NOX2 levels, severity, and subsequent clinical outcomes of severe traumatic brain injury (sTBI).

**Methods:**

In this prospective cohort study, serum NOX2 levels were measured in 123 patients and 123 controls. The Glasgow Coma Scale (GCS) scores and Rotterdam computed tomography (CT) classifications were applied for assessing injury severity. A poor prognosis was considered if the Glasgow Outcome Scale Extended (GOSE) score was 4 or below at 180 days post‐injury.

**Results:**

STBI patients exhibited markedly enhanced serum NOX2 levels relative to healthy controls, and serum NOX2 levels were independently linked to Rotterdam CT classifications and GCS scores. Serum NOX2 levels effectively identified individuals at risk of death or poor prognosis at 180‐day after sTBI. When compared to GCS scores and Rotterdam CT classifications, its predictive power was comparable. When the three variables were utilized together, the model's predictive ability was significantly higher than when they were independently used.

**Conclusions:**

NOX2 might be used as a potential biomarker to assess the severity of sTBI and foretell its outcome, since elevated serum NOX2 levels are significantly linked to increasing severity, 180‐day mortality, and poor prognosis after sTBI.

## Graphical abstract

This prospective cohort study found that serum NOX2 levels were closely related to the disease severity of sTBI patients and could predict the 180‐day mortality and poor prognosis of the patients.

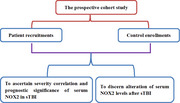

**Abbreviations**
95% CI: 95% confidence interval
AUC: area under the curve
CT: computed tomography
GCS: Glasgow coma scale
NADPH: nicotinamide adenine dinucleotide phosphate.
NOX: nicotinamide adenine dinucleotide phosphate oxidase 2
ROS: reactive oxygen species
sTBI: severe traumatic brain injury
TBI: traumatic brain injury
VIF: variation inflation factor

## 1 Introduction

Traumatic brain injury (TBI), a prevalent and serious disorder in the neurosurgical profession, is marked by brain dysfunction and histopathological abnormalities as a result of external force (Kaur and Sharma 2018). Clinically, Glasgow Coma Scale (GCS) scores of 8 or less indicate severe traumatic brain injury (sTBI), the most serious kind of TBI (Mostert et al. 2022). Factors, such as inflammatory reactions, oxidative stress, mitochondrial malfunction, calcium overload, and the synthesis of excitatory neurotransmitters, induce secondary brain damage, which remains throughout the illness course after primary brain injury. Neurological impairments or death may result from these processes, which cause malfunction and necrosis at the injury site, as well as surrounding brain tissues (Sulhan et al. 2020, Orr et al. 2024). The prognosis of sTBI is often predicted using GCS scores and Rotterdam computed tomography (CT) classifications (Ranganathan et al. 2019, Zeng et al. 2023, Fujimoto et al. 2016). In recent decades, biomarkers such as Tau proteins, netrin‐1, and nitric oxide metabolite have been the subject of extensive research due to their unique prognostic values in acute brain injury (Liliang et al. 2010, Kandasamy et al. 2013, Xie et al. 2021).

Nicotinamide adenine dinucleotide phosphate (NADPH) oxidase 2 (NOX2) is the main contributor to the production of reactive oxygen species (ROS) in brain tissues (Wang et al. 2022, Wang et al. 2020). Mice suffering from acute cerebral ischemia had abnormally high NOX2 levels in their brain tissues, and reducing or inhibiting NOX2 expression significantly lessened the extent of the infarction, attenuated damage to the blood‐brain barrier (BBB), and improved neurological function (Genovese et al. 2011, Chen et al. 2009). Also, diminishing damage volume, improving motor and cognitive abilities, and significantly lowering ROS expressions in brain tissues were all seen in TBI animals given NOX2 inhibitors (Chandran et al. 2018). Moreover, patients with aneurysmal subarachnoid hemorrhage (aSAH) had significantly higher serum NOX2 levels, which were closely associated with a higher risk of delayed cerebral ischemia and a worse prognosis for patients (Wu et al. 2023). Based on this evidence, NOX2 may be a potential bioindicator for acute brain damage. This research aims to determine whether serum NOX2 levels are linked to illness severity, 180‐day mortality, and worse prognosis among individuals with sTBI.

## 2 Materials and Methods

### 2.1 Study Design and Participant Recruitments

**FIGURE 1:**
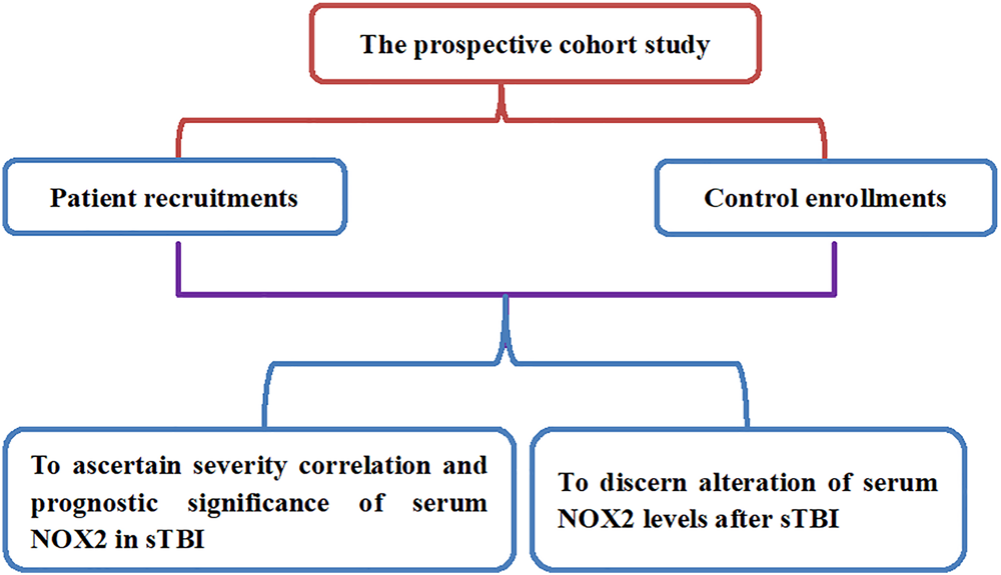
Diagrammatical sketch showing study design. In accordance with the prespecified inclusion and exclusion criteria, patients with sTBI and controls were enrolled for determining variation of serum NOX2 levels and assessing its prognostic role in sTBI. NOX2 means nicotinamide adenine dinucleotide phosphate oxidase 2; sTBI, severe traumatic brain injury.

In this prospective cohort study at Lishui People's Hospital (Lishui, China), all patients with sTBI were consecutively enrolled between January 2020 and January 2024. Inclusion criteria were as follows: (1) GCS score equal to or less than 8, (2) admission into hospital within 12 h following trauma, and (3) isolated and blunt head trauma. Exclusion criteria were in the following: (1) individuals who have not yet reached the legal age of majority; (2) information that is incomplete or missing; (3) appointments that are not followed up as scheduled; (4) refusal to participate; (5) inability to obtain blood samples that have been hemolyzed or lipohemolyzed; (6) recent surgery or infection; (7) neurological disorders (such as ischemic stroke, cerebral hemorrhage, or traumatic brain injury); and (8) pregnancy or the existence of other serious conditions, like uremia, liver cirrhosis, heart failure, liver failure, or malignant tumors. Between July 2023 and January 2024, healthy controls were recruited from the Physical Examination Center at Lishui People's Hospital. Controls were free from some chronic diseases, such as hypertension, diabetes mellitus, hyperlipidemia, and more, and also had normal ranges in some conventional tests, like blood leukocyte counts, blood electrolyte levels, blood glucose levels, chest X‐ray, electrocardiogram, and so forth. According to Figure 1, serum NOX2 levels were detected in patients and controls, and patients were followed up until death or completion of 180 days after injury. The research followed all the guidelines laid forth in the Declaration of Helsinki, and approval to the study was given from the Lishui People's Hospital Ethics Committee (No. 2020‐001, 2020‐002). Since the patients included in this study had impaired consciousness, their legal representatives were authorized to sign the written informed consent. Additionally, the healthy controls provided written informed consent.

### 2.2 Variables

The collected information included a wide range of factors, covering gender, age, medications, trauma causes, and any previous health issues. At the time of admission, some data, such as vital signs, trauma severity ratings, and radiological characteristics, were recorded. Rotterdam CT classifications and GCS scores were used to assess severity. The radiological abnormalities encompassed midline shift, subdural bleeding, epidural hemorrhage, subarachnoid hemorrhage, intraventricular hemorrhage, intracerebral hemorrhage, brain contusion, and pneumocephalus. The Glasgow Outcome Scale Extended (GOSE) was employed for evaluation of their neurological prognosis. The GOSE score 1 means dead; 2, vegetative state; 3, lower severe disability; 4, upper severe disability; 5, lower moderate disability; 6, upper moderate disability; 7, lower good recovery; and 8, upper good recovery (Wilson et al. 2021). Using structured interviews, functional assessment was completed via telephone by the trained personnel at 180 days after head trauma. Patient prognosis was poor when the GOSE scores were between 1–4 (Banoei et al. 2023). Poor prognosis and all‐cause death within 180 days following injury were considered as the two outcome variables of interest.

### 2.3 Immune Analysis

Patients admitted to the hospital with sTBI and healthy controls had normal physical tests, during which peripheral venous blood samples were taken. Typical methods were used to evaluate standard laboratory indicators, including blood leukocyte counts, glucose levels, and potassium levels. Purified serum was obtained by spinning peripheral venous blood at 3000 × g for 10 min, followed by storage at ‐80°C until use in further analysis. Shanghai Jianglai Biotechnology Co., Ltd. (China) supplied the enzyme‐linked immunosorbent assay kit used for determination of the serum NOX2 levels. The limit of detection was 0.075 ng/mL, and the detection range was from 0.156 to 10 ng/mL. There was less than 15% variation across the plates and less than 10% variation within each plate. Each sample underwent the procedure twice, with the mean value serving as the end outcome. The testing technician had no prior knowledge of the patient's medical history.

### 2.4 Statistical Analysis

To conduct our data analysis, we used MedCalc 9.6.4.0 (MedCalc Software, Mariakerke, Belgium) and SPSS 26.0 (IBM Corporation, Armonk, New York, USA). The counter‐graphs were created with GraphPad Prism 8.0 (GraphPad Software Inc., La Jolla, California, United States) and R software (version 4.2.4; https://www.r‐project.org). Normally distributed continuous variables were analyzed using the t‐test and are presented as mean plus standard deviation. Non‐normally distributed variables were analyzed using the Mann‐Whitney U test and are shown as median (25th‐75th percentiles). Categorical variables are reported using counts (percentages). To compare two sets of categorical data, we used either Fisher's exact or χ^2^ test, as appropriate. The Kruskal‐Wallis H test was done to compare non‐normally distributed continuous variables among several groups. The Bonferroni method was employed for correction of multiple comparisons as deemed appropriate. The Spearman correlation test was employed to ascertain the link between each variable and the serum NOX2 levels and GOSE scores. Using a multivariate linear regression model, we were able to screen components that exhibit a non‐independent relationship with serum NOX2 levels and GOSE scores. Multifactorial logistic regression was used to compare patients with good prognoses and those with poor prognoses, as well as those who survived and those who did not. Our goal was to explore factors that promote poor prognosis, as well as attributes that lead to death 180 days after a sTBI. We presented the data using odds ratio (OR) and a 95% confidence interval (CI). Patients with sTBI had their serum NOX2 levels analyzed using a receiver operating characteristic (ROC) curve to determine their predictive value for 180‐day mortality and poor prognoses. Once the area under the curve (AUC) results were generated, they were compared using the Z‐test. The relationships between mortality, poor prognosis and other variables, as measured by serum NOX2 levels, GCS scores, and Rotterdam CT classifications, were shown in a column‐line graph. Utilizing a calibration curve model, the reliability of serum NOX2 levels as an indicator of death and poor prognosis was evaluated. Using a decision curve analysis, we checked how well the prediction model worked in a clinical setting. *P* < 0.05 was set as the significance standard.

## 3 Results

### 3.1 Study Participant Features

Initially, 152 sTBI patients were consecutively enrolled; however, 29 participants were eliminated according to the prespecified criteria, resulting in the inclusion of 123 patients in our final investigation (Supplemental Figure 1). An equal number of healthy controls (n = 123) were recruited for comparison. Table 1 presents the fundamental profiles of the study participants who were initially enrolled and those who were ultimately eligible. No notable differences were found between the patients who were initially enrolled and those who were finally deemed eligible across all variables (all *P* > 0.05). Among 123 healthy controls, there were 67 males and 56 females, with a median age of 51 years (lower‐upper quartiles, 45–59 years); and 21 (17.1%) individuals were cigarette smokers and 33 (26.8%) subjects were alcohol consumers. The baseline statistics for age, gender, smoking, and alcohol use showed that the final qualified patients and healthy controls were similar (all *P* > 0.05).

**TABLE 1:** Differences in demographic data and vascular risk factors between patients and controls.

Variables	Initially enrolled patients (n=152)	Finally eligible patients (n=123)	*P* value
Gander (male/female)	74/78	57/66	0.234
Age (years)	49 (34‐60)	49 (35‐61)	0.299
Cigarette smoking	40 (26.3%)	32 (26.0%)	0.863
Alcohol consumption	45 (29.6%)	39 (31.7%)	0.242
Hypertension	39 (25.7%)	32 (26.0%)	0.835
Diabetes mellitus	21 (13.8%)	18 (14.6%)	0.547
Hyperlipidemia	44 (28.9%)	35 (28.5%)	0.783
Hospital admission time (h)	4.2 (2.6‐5.6)	4.3 (2.8‐5.9)	0.169
Blood‐sampling time (h)	5.2 (3.6‐6.8)	5.4 (3.6‐6.8)	0.154
Systolic arterial pressure (mmHg)	114 (96‐131)	112 (94‐128)	0.264
Diastolic arterial pressure (mmHg)	72 (63‐82)	72 (63‐82)	0.877
Traumatic causes			0.613
car/motorcycle	80 (52.6%)	65 (52.8%)	
fall/jump	49 (32.2%)	41 (33.3%)	
others	23 (15.1%)	17 (13.8%)	
GCS scores	4 (4‐6)	5 (4‐7)	0.839
Rotterdam CT classification	4 (4‐5)	4 (4‐5)	0.957
Midline shift > 5 mm	85 (55.9%)	71 (57.7%)	0.357
Abnormal cisterns	112 (73.7%)	91 (74.0%)	0.863
Epidural hematoma	82 (53.9%)	67 (54.5%)	0.789
Subdural hematoma	89 (58.6%)	69 (56.1%)	0.206
Subarachnoid hemorrhage	104 (68.4%)	82 (66.7%)	0.338
Intraventricular hemorrhage	22 (14.5%)	18 (14.6%)	0.908
Intracerebral hematoma	81 (53.3%)	67 (54.5%)	0.547
Brain contusion	108 (71.1%)	87 (70.7%)	0.857
Pneumocephalus	56 (36.8%)	47 (38.2%)	0.471
Blood leukocyte count (×10^9^/L)	9.1 (7.3‐10.1)	9.3 (7.2‐10.2)	0.482
Blood CRP levels (mg/L)	6.0 (2.2‐11.6)	6.0 (2.0‐11.5)	0.069
Blood potassium levels (mmol/L)	3.66 (3.43‐3.95)	3.64 (3.41‐3.93)	0.173
Blood glucose levels (mmol/l)	8.1 (5.8‐11.8)	7.9 (5.7‐12.5)	0.729

Note: Quantitative data were reported as medians with upper and lower quartiles or means ± standard deviations, as appropriate. Qualitative data were presented as counts (proportions). Intergroup comparisons were performed using the χ2 test or Fisher's exact test for qualitative data and the Mann‐Whitney U‐test or t‐test for quantitative data.

Abbreviations: CRP, C‐reactive protein; GCS, Glasgow coma scale; CT, computed tomography.

### 3.2 Correlation Analysis Between Serum NOX2 Levels and Disease Severity

**FIGURE 2:**
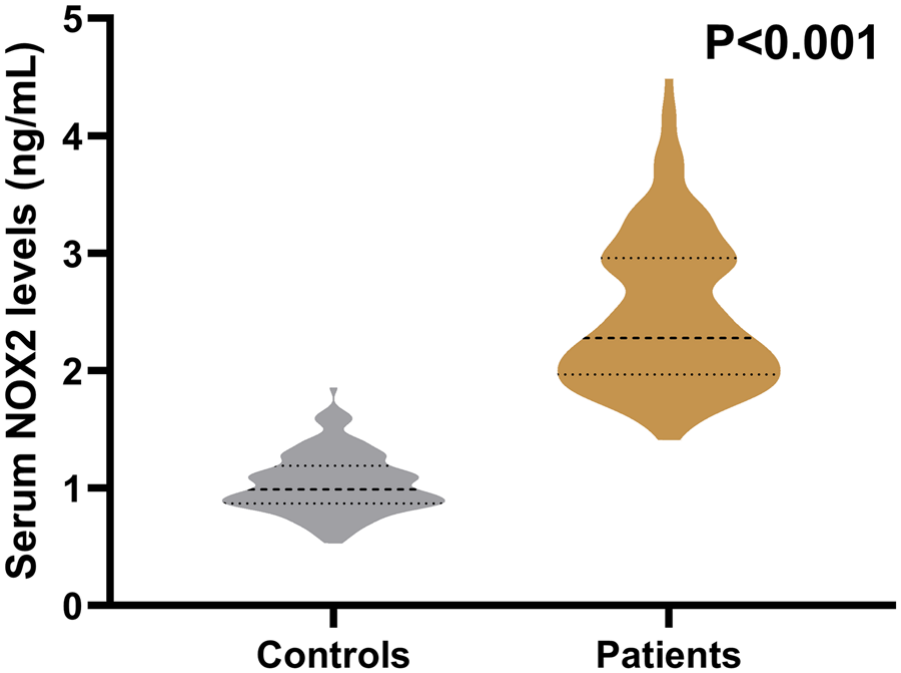
Comparison of serum NOX2 levels in individuals with sTBI and controls. As shown in the boxplot, subjects with sTBI had significantly higher serum NOX2 levels when compared to controls (*P* < 0.001). NOX2 stands for nicotinamide adenine dinucleotide phosphate oxidase 2; sTBI, severe traumatic brain injury.

Compared to the control group, all injured brain patients had significantly higher serum NOX2 levels (*P* < 0.001; Figure 2). Serum NOX2 levels were significantly related to categorical (*P* < 0.001; Supplemental Figure 2) and continuous GCS scores (*P* < 0.001; Supplemental Figure 3), as well as substantially correlated with categorical (*P* < 0.001; Supplemental Figure 4) and continuous Rotterdam CT classifications (*P* < 0.001; Supplemental Figure 5). Table 2 shows that blood glucose levels, blood leukocyte count, blood C‐reactive protein (CRP) levels, abnormal cisterns, midline shift > 5 mm, intraventricular hemorrhage, intracerebral hemorrhage, subdural hematoma, epidural hematoma, and subdural hematoma were markedly correlated with serum NOX2 levels (all *P* < 0.05). Also, all of the findings were supported by the univariate linear regression analysis (all *P* < 0.05; Table 2). Table 3 summarizes the findings from the multivariate linear regression model, demonstrating an independent relationship between serum NOX2 levels and both GCS scores and Rotterdam CT classifications (all *P* < 0.05).

**TABLE 2:** Factors correlated with serum nicotinamide adenine dinucleotide phosphate oxidase 2 levels after severe traumatic brain injury

			Univariate linear regression	
	ρ	*P* value	β (95% CI)	*P* value
Gender (male/female)	−0.072	0.430	−0.093 (‐0.326‐0.141)	0.434
Age (y)	0.144	0.112	0.007 (0.000‐0.015)	0.064
Current cigarette smoking	0.096	0.291	0.157 (‐0.108‐0.422)	0.242
Alcohol abuse	0.089	0.329	0.164 (‐0.086‐0.413)	0.196
Hypertension	0.087	0.339	0.077 (‐0.189‐0.343)	0.569
Diabetes mellitus	0.143	0.115	0.289 (‐0.038‐0.616)	0.082
Hyperlipidemia	0.095	0.297	0.144 (‐0.116‐0.404)	0.274
Hospital admission time (h)	−0.152	0.094	−0.045 (‐0.095‐0.005)	0.079
Blood‐sampling time (h)	−1.119	0.191	−0.037 (‐0.086‐0.012)	0.136
Systolic arterial pressure (mmHg)	−0.157	0.083	−0.003 (‐0.008‐0.001)	0.150
Diastolic arterial pressure (mmHg)	−0.042	0.645	−0.001 (‐0.008‐0.007)	0.879
Traumatic causes	−0.118	0.194	−0.109 (‐0.271‐0.053)	0.184
GCS scores	−0.565	<0.001	−0.236 (‐0.297–0.174)	<0.001
Rotterdam CT classification	0.590	<0.001	0.407 (0.308‐0.506)	<0.001
Midline shift > 5 mm	0.229	0.011	0.301 (0.071‐0.532)	0.011
Abnormal cisterns	0.224	0.013	0.361 (0.103‐0.619)	0.007
Epidural hematoma	0.302	0.001	0.378 (0.153‐0.603)	0.001
Subdural hematoma	0.227	0.012	0.289 (0.059‐0.519)	0.014
Subarachnoid hemorrhage	0.092	0.311	0.195 (‐0.051‐0.440)	0.119
Intraventricular hemorrhage	0.208	0.021	0.494 (0.175‐0.812)	0.003
Intracerebral hematoma	0.209	0.020	0.313 (0.085‐0.542)	0.008
Brain contusion	0.114	0.210	0.172 (‐0.083‐0.427)	0.185
Pneumocephalus	0.029	0.748	−0.022 (‐0.263‐0.219)	0.856
Blood leukocyte count (×10^9^/L)	0.320	<0.001	0.070 (0.023‐0.117)	0.004
Blood CRP levels (mg/L)	0.252	0.005	0.025 (0.008‐0.042)	0.005
Blood potassium levels (mmol/L)	−0.004	0.962	−0.034 (‐0.280‐0.212)	0.786
Blood glucose levels (mmol/l)	0.253	0.005	0.045 (0.015‐0.075)	0.003

Note: Correlations were analyzed using the Spearman test. 95% CI, 95% confidence interval.

Abbreviations: CT, computerized tomography; CRP, C‐reactive protein; GCS, Glasgow coma scale.

**TABLE 3:** Correlation between serum nicotinamide adenine dinucleotide phosphate oxidase 2 levels and other variables using multivariate linear regression analysis in severe traumatic brain injury

	β (95% CI)	VIF	*P* value
GCS scores	−0.108 (‐0.197–0.019)	2.412	0.018
Rotterdam CT classification	0.173 (0.011‐0.335)	2.916	0.037
Midline shift > 5 mm	0.061 (‐0.143‐0.265)	1.260	0.556
Abnormal cisterns	0.058 (‐0.167‐0.282)	1.203	0.613
Epidural hematoma	0.060 (‐0.160‐0.281)	1.487	0.587
Subdural hematoma	0.103 (‐0.097‐0.303)	1.218	0.308
Intraventricular hemorrhage	0.157 (‐0.120‐0.434)	1.188	0.264
Intracerebral hematoma	0.125 (‐0.075‐0.324)	1.216	0.219
Blood leukocyte count (×10^9^/l)	0.027 (‐0.014‐0.068)	1.223	0.198
Blood CRP levels (mg/L)	0.009 (‐0.007‐0.024)	1.236	0.269
Blood glucose levels (mmol/l)	0.010 (‐0.017‐0.038)	1.360	0.457

Note: Correlations were done using a multivariate linear regression model in severe traumatic brain injury.

Abbreviations: CRP, C‐reactive protein; CT, computerized tomography; GCS, Glasgow coma scale; VIF, variance inflation factor; 95% CI, 95% confidence interval.

### 3.3 Link Between Serum NOX2 Levels and Post‐sTBI GOSE Scores

As shown in Table 4, the 180‐day GOSE scores of sTBI patients were significantly correlated with serum NOX2 levels, GCS scores, Rotterdam CT classifications, midline shift >5 mm, acute hydrocephalus, epidural hemorrhage, subdural hemorrhage, intraventricular hemorrhage, blood leukocyte counts, blood CRP levels, blood glucose levels, and serum NOX2 levels (all *P* < 0.05). Similar results were confirmed by univariate linear regression analysis (all *P* < 0.05; Table 4). As shown in Table 5, GCS scores, Rotterdam CT classifications, and serum NOX2 levels were independently associated with the 180‐day GOSE scores after injury (all *P* < 0.05). Next, the patients' GOSE was applied for grouping and 29, 10, 7, 13, 22, 16, 15, and 11 cases had the scores from 1 to 8 separately. A strong association between the scores and serum NOX2 levels was found regardless of the GOSE as a categorical (*P* < 0.001; Supplemental Figure 6) or continuous variable (*P* < 0.001; Supplemental Figure 7).

**TABLE 4:** Factors correlated with extended Glasgow Outcome Scale scores after severe traumatic brain injury

			Univariate linear regression	
	ρ	*P* value	β (95% CI)	*P* value
Gender (male/female)	0.060	0.510	0.178 (‐0.681‐1.037)	0.683
Age (y)	0.004	0.965	−0.002 (‐0.030‐0.027)	0.911
Current cigarette smoking	0.001	0.993	−0.023 (‐1.000‐0.954)	0.963
Alcohol abuse	−0.043	0.634	−0.308 (‐1.227‐0.612)	0.509
Hypertension	−0.057	0.534	−0.319 (‐1.294‐0.657)	0.519
Diabetes mellitus	−0.069	0.449	−0.471 (‐1.682‐0.739)	0.442
Hyperlipidemia	−0.103	0.258	−0.529 (‐1.475‐0.416)	0.270
Hospital admission time (h)	0.095	0.298	0.133 (‐0.052‐0.318)	0.157
Blood‐sampling time (h)	0.072	0.432	0.105 (‐0.074‐0.285)	0.247
Systolic arterial pressure (mmHg)	0.140	0.123	0.011 (‐0.007‐0.028)	0.229
Diastolic arterial pressure (mmHg)	0.119	0.189	0.015 (‐0.013‐0.042)	0.294
Traumatic causes	−0.015	0.866	−0.153 (‐0.750‐0.444)	0.613
GCS scores	0.618	<0.001	0.949 (0.734‐1.163)	<0.001
Rotterdam CT classification	−0.627	<0.001	−1.629 (‐1.973–1.285)	<0.001
Midline shift > 5 mm	−0.245	0.006	−1.191 (‐2.032–0.350)	0.006
Abnormal cisterns	−0.230	0.010	−1.244 (‐2.196–0.293)	0.011
Epidural hematoma	−0.246	0.006	−1.206 (‐2.040–0.373)	0.005
Subdural hematoma	−0.192	0.034	−0.867 (‐1.717–0.017)	0.046
Subarachnoid hemorrhage	−0.111	0.221	−0.561 (‐1.465‐0.343)	0.222
Intraventricular hemorrhage	−0.204	0.023	−1.383 (‐2.570–0.195)	0.023
Intracerebral hematoma	−0.150	0.097	−0.780 (‐1.630‐0.069)	0.072
Brain contusion	−0.196	0.030	−1.002 (‐1.927–0.077)	0.034
Pneumocephalus	0.043	0.639	0.273 (‐0.608‐1.154)	0.541
Blood leukocyte count (×10^9^/L)	−0.182	0.044	−0.196 (‐0.373–0.018)	0.031
Blood CRP levels (mg/L)	−0.260	0.004	−0.071 (‐0.134–0.007)	0.030
Blood potassium levels (mmol/L)	0.029	0.704	−0.058 (‐0.960‐0.843)	0.898
Blood glucose levels (mmol/l)	−0.343	<0.001	−0.191 (‐0.299–0.082)	0.001
Serum NOX2 levels (ng/mL)	−0.602	<0.001	−2.164 (‐2.697–1.631)	<0.001

Note: Correlations were analyzed using the Spearman test.

Abbreviations: C‐reactive protein; CT, computerized tomography; GCS, Glasgow coma scale; CRP, NOX2, nicotinamide adenine dinucleotide phosphate oxidase 2; 95% CI, 95% confidence interval.

**TABLE 5:** Correlations between extended Glasgow Outcome Scale scores and other variables using multivariate linear regression analysis in severe traumatic brain injury

	β (95% CI)	VIF	*P* value
GCS scores	0.325 (0.012‐0.639)	2.535	0.042
Rotterdam CT classification	−0.828 (‐1.393–0.263)	3.028	0.004
Midline shift > 5 mm	−0.475 (‐1.175‐0.226)	1.262	0.182
Abnormal cisterns	0.016 (‐0.753‐0.786)	1.200	0.966
Epidural hematoma	0.041 (‐0.700‐0.781)	1.432	0.914
Subdural hematoma	−0.166 (‐0.858‐0.527)	1.244	0.636
Intraventricular hemorrhage	−0.010 (‐0.966‐0.955)	1.201	0.983
Brain contusion	−0.358 (‐1.079‐0.364)	1.136	0.328
Blood leukocyte count (×10^9^/l)	0.053 (‐0.089‐0.195)	1.236	0.458
Blood CRP levels (mg/L)	0.008 (‐0.044‐0.061)	1.251	0.755
Blood glucose levels (mmol/l)	−0.024 (‐0.120‐0.072)	1.379	0.614
Serum NOX2 levels (ng/mL)	−0.904 (‐1.545–0.263)	1.826	0.006

Note: Correlations were done using multivariate linear regression analysis in severe traumatic brain injury.

Abbreviations: CRP, C‐reactive protein; CT, computerized tomography; GCS, Glasgow coma scale; NOX2, nicotinamide adenine dinucleotide phosphate oxidase 2; 95% CI, 95% confidence interval; VIF, variance inflation factor.

### 3.4 Serum NOX2 Levels and 180‐Day Death Post‐sTBI

**FIGURE 3:**
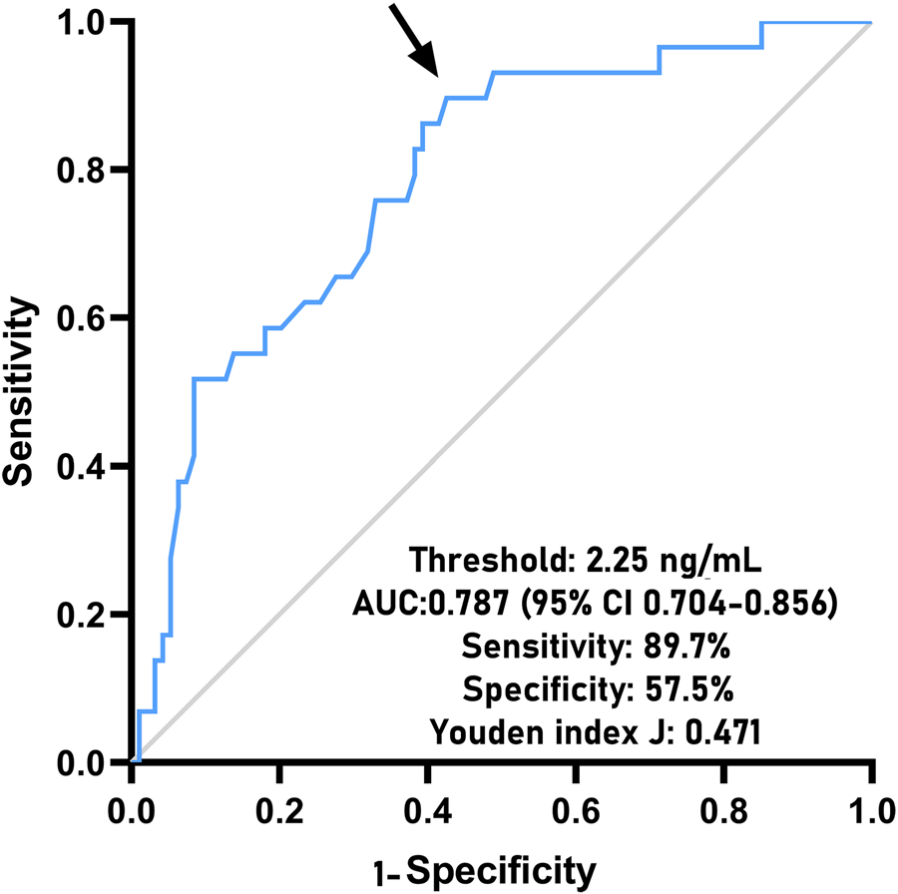
Predictive ability of serum NOX2 levels for 180‐day death after sTBI. Under the receiver operating characteristic curve, serum NOX2 levels efficiently anticipated death at 180 days post‐sTBI, and the threshold value was chosen using the Youden method for foretelling death with the maximum Youden index. NOX2 denotes nicotinamide adenine dinucleotide phosphate oxidase 2; sTBI, severe traumatic brain injury; AUC, area under curve; 95% CI, 95% confidence interval.

A total of 29 patients were deceased within 180 days following head trauma. Serum NOX2 levels were markedly elevated among non‐survivors versus survivors (*P* < 0.001; Supplemental Figure 8). ROC curve assessment indicated that serum NOX2 levels over 2.25 ng/mL substantially distinguished the mortality risk in sTBI patients (Figure 3). Moreover, in contrast to survivors, non‐survivors demonstrated significantly reduced GCS scores, elevated Rotterdam CT classifications, increased blood leukocyte counts, heightened blood CRP levels, augmented blood glucose levels, elevated serum NOX2 levels, and a greater prevalence of midline shift >5 mm, intracerebral pooling abnormalities, epidural hematoma, and intraventricular hemorrhage (all *P* < 0.05; Table 6). Univariate logistic regression analysis demonstrated significant disparities between survivors and non‐survivors regarding the above‐mentioned variables (all *P* < 0.05; Table 7). The inclusion of these ten variables in the binary logistic regression model revealed that GCS scores, Rotterdam CT classifications, and serum NOX2 levels were stand‐alone regulators of 180‐day mortality following sTBI (all *P* < 0.05; Table 7).

**TABLE 6:** Factors associated with 180‐day mortality in severe traumatic brain injury

	The alive (n=94)	The death (n=29)	*Z/χ^2^*	*P* value
Gender (male/female)	46/48	11/18	1.079	0.299
Age (y)	49 (35‐61)	54 (34‐63)	−0.414	0.679
Current cigarette smoking	25 (26.6%)	7 (24.1%)	0.070	0.792
Alcohol abuse	29 (30.9%)	10 (34.5%)	0.135	0.713
Hypertension	23 (24.5%)	9 (31.0%)	0.496	0.481
Diabetes mellitus	12 (12.8%)	6 (20.7%)	1.114	0.291
Hyperlipidemia	26 (27.7%)	8 (27.6%)	0.124	0.725
Hospital admission time (h)	4.3 (2.8‐6.0)	4.1 (2.8‐4.6)	−1.478	0.139
Blood‐sampling time (h)	5.6 (3.6‐6.9)	4.6 (3.7‐6.1)	−1.392	0.164
Systolic arterial pressure (mmHg)	116 (95‐134)	109 (92‐114)	−1.791	0.073
Diastolic arterial pressure (mmHg)	73 (63‐84)	66 (60‐74)	−1.550	0.121
Traumatic causes			3.355	0.187
Automobile/motorcycle	48 (51.1%)	17 (58.6%)	—	—
Fall/jump	35 (37.2%)	6 (20.7%)	—	—
Others	11 (11.7%)	6 (20.7%)	—	—
GCS scores	5 (4‐7)	4 (3‐5)	−4.853	<0.001
Rotterdam CT classification	4 (4‐5)	6 (5‐6)	−5.119	<0.001
Midline shift > 5 mm	49 (52.1%)	24 (75.8%)	5.116	0.024
Abnormal cisterns	65 (69.1%)	26 (89.7%)	4.842	0.028
Epidural hematoma	48 (51.1%)	19 (65.5%)	1.867	0.172
Subdural hematoma	51 (54.3%)	18 (62.1%)	0.549	0.459
Subarachnoid hemorrhage	61 (64.9%)	21 (72.4%)	0.564	0.453
Intraventricular hemorrhage	10 (10.6%)	8 (27.6%)	5.096	0.024
Intracerebral hematoma	46 (48.9%)	21 (72.4%)	4.926	0.026
Brain contusion	66 (70.2%)	21 (72.4%)	0.052	0.820
Pneumocephalus	36 (38.3%)	11 (37.9%)	0.001	0.972
Blood leukocyte count (×10^9^/l)	8.9 (6.8‐9.9)	9.7 (7.9‐11.2)	−2.399	0.016
Blood CRP concentrations (mg/L)	4.4 (2.0‐11.0)	8.9 (3.4‐13.3)	−2.484	0.013
Blood potassium concentrations (mmol/L)	3.63 (3.42‐3.94)	3.66 (3.32‐3.94)	−0.030	0.976
Blood glucose levels (mmol/l)	7.1 (5.3‐12.0)	9.5 (7.2‐13.3)	−2.464	0.014
Serum NOX2 levels (ng/mL)	2.14 (1.87‐2.87)	3.11 (2.44‐3.35)	−4.660	<0.001

*Note*: Qualitative variables were presented as counts (percentages) and were compared for intergroup difference using the chi‐square test or Fisher's exact test, as appropriate. Quantitative variables were summarized as medians (upper ‐ lower quartiles) or the means ± standard deviations, as appropriate. Intergroup comparisons were done using an unpaired Student's t‐test or Mann‐Whitney U test where appropriate.

Abbreviations: CRP, C‐reactive protein; CT, computerized tomography; GCS, Glasgow coma scale; NOX2, nicotinamide adenine dinucleotide phosphate oxidase 2.

**TABLE 7:** Univariate and multivariate binary logistic regression analysis of predictors for 180‐day death after severe traumatic brain injury

	Univariate analysis		Multivariate analysis	
	Odds ratio (95% *CI*)	*P* value	Odds ratio (95% *CI*)	*P* value
Gender (male/female)	1.568 (0.669‐3.677)	0.301	—	—
Age (y)	1.005 (0.978‐1.033)	0.720	—	—
Current cigarette smoking	0.878 (0.334‐2.307)	0.792	—	—
Alcohol abuse	1.180 (0.488‐2.850)	0.713	—	—
Hypertension	1.389 (0.556‐3.474)	0.482	—	—
Diabetes mellitus	1.783 (0.603‐5.268)	0.296	—	—
Hyperlipidemia	0.996 (0.393‐2.529)	0.994	—	—
Hospital admission time (h)	0.841 (0.688‐1.027)	0.089	—	—
Blood‐sampling time (h)	0.858 (0.710‐1.036)	0.111	—	—
Systolic arterial pressure (mmHg)	0.986 (0.968‐1.003)	0.114	—	—
Diastolic arterial pressure (mmHg)	0.983 (0.957‐1.010)	0.220	—	—
Traumatic causes		0.199	—	—
Automobile/motorcycle	1			
Fall/jump	0.649 (0.208‐2.027)	0.457	—	—
Others	0.314 (0.084‐1.175)	0.085	—	—
GCS scores	0.401 (0.263‐0.612)	<0.001	0.516 (0.275‐0.968)	0.039
Rotterdam CT classification	4.107 (2.287‐7.377)	<0.001	2.913 (1.132‐7.496)	0.027
Midline shift > 5 mm	2.885 (1.125‐7.403)	0.027	1.521 (0.435‐5.315)	0.511
Abnormal cisterns	3.867 (1.083‐13.806)	0.037	1.918 (0.362‐10.166)	0.444
Epidural hematoma	1.821 (0.766‐4.329)	0.175	—	—
Subdural hematoma	1.380 (0.588‐3.237)	0.459	—	—
Subarachnoid hemorrhage	1.420 (0.567‐3.556)	0.454	—	—
Intraventricular hemorrhage	3.200 (1.125‐9.103)	0.029	1.400 (0.312‐6.275)	0.660
Intracerebral hematoma	2.519 (1.013‐6.245)	0.047	1.410 (0.394‐5.050)	0.598
Brain contusion	1.114 (0.441‐2.813)	0.820	—	—
Pneumocephalus	0.985 (0.418‐2.321)	0.972	—	—
Blood leukocyte count (×10^9^/L)	1.203 (1.004‐1.441)	0.046	1.108 (0.856‐1.435)	0.435
Blood CRP concentrations (mg/L)	1.063 (1.001‐1.128)	0.046	0.910 (0.822‐1.008)	0.071
Blood potassium concentrations (mmol/L)	1.220 (0.515‐2.891)	0.651	—	—
Blood glucose levels (mmol/l)	1.122 (1.006‐1.250)	0.038	0.995 (0.834‐1.186)	0.952
Serum NOX2 levels (ng/mL)	4.679 (2.243‐9.760)	<0.001	3.604 (1.162‐11.182)	0.026

*Note*: Results were presented as odds ratios (95% confidence interval) using the univariate and multivariate binary logistic regression analysis. 95% CI indicates 95% confidence interval.

Abbreviations: CRP, C‐reactive protein; CT, computerized tomography; GCS, Glasgow coma scale; NOX2, nicotinamide adenine dinucleotide phosphate oxidase 2.

**FIGURE 4:**
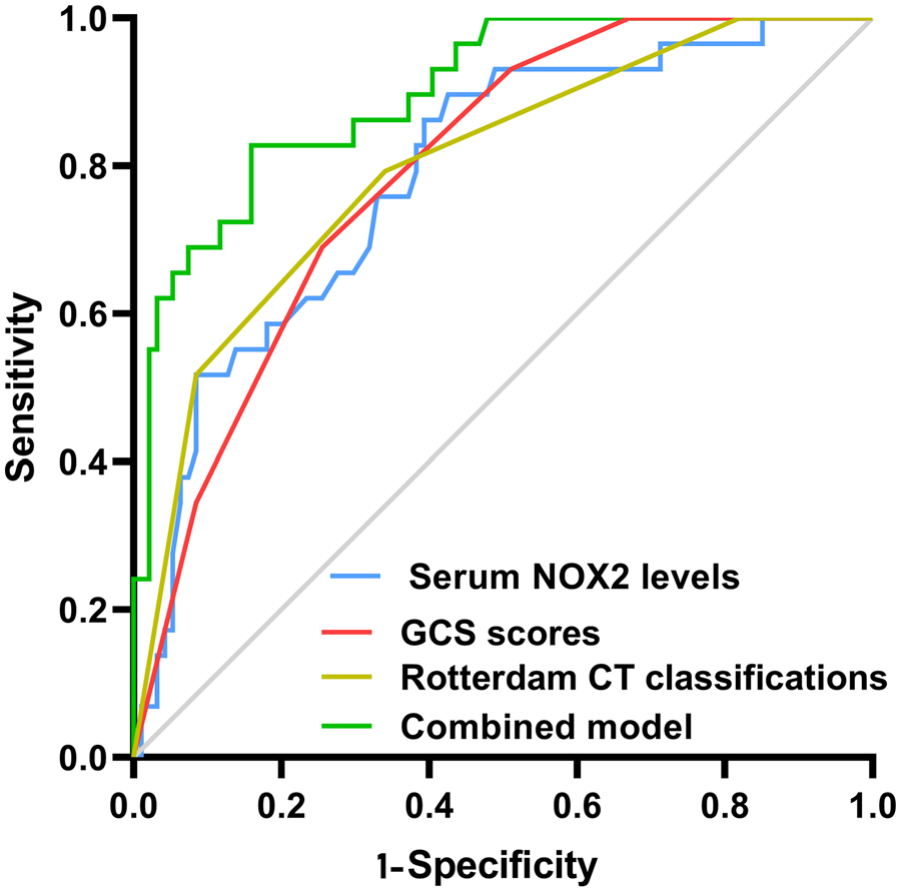
Assessment of predictive capability for post‐sTBI 180‐day mortality between serum NOX2 levels, GCS scores, Rotterdam CT classifications, and the combined model. Under the ROC curve, serum NOX2 levels had predictive ability comparable to those of GCS scores and Rotterdam CT classifications (both *P* > 0.05). The preceding three variables were integrated to form a prediction model. The combined model outperformed their alone use (all *P* < 0.05). NOX2 signifies nicotinamide adenine dinucleotide phosphate oxidase 2; GCS, Glasgow coma scale; CT, computerized tomography; sTBI, severe traumatic brain injury; ROC, receiver operating characteristic.

**FIGURE 5:**
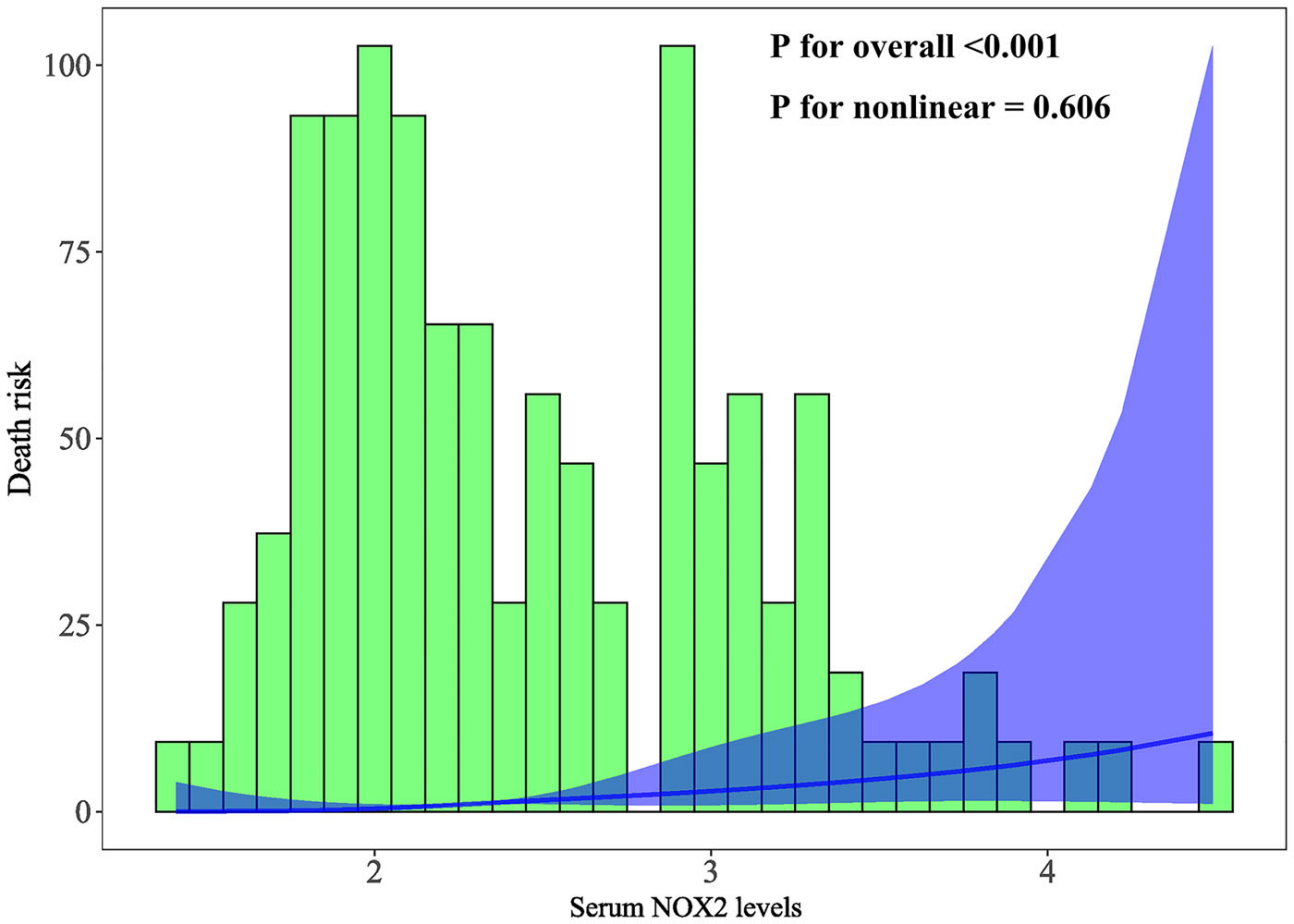
Linear relationship between serum NOX2 levels and the probability of dying within 180 days following sTBI. By plotting restricted cubic spline, serum NOX2 levels showed a strong linear relationship with the probability of death at 180 days after sTBI (*p* > 0.05). NOX2 stands for nicotinamide adenine dinucleotide phosphate oxidase 2; sTBI, severe traumatic brain injury.

**FIGURE 6:**
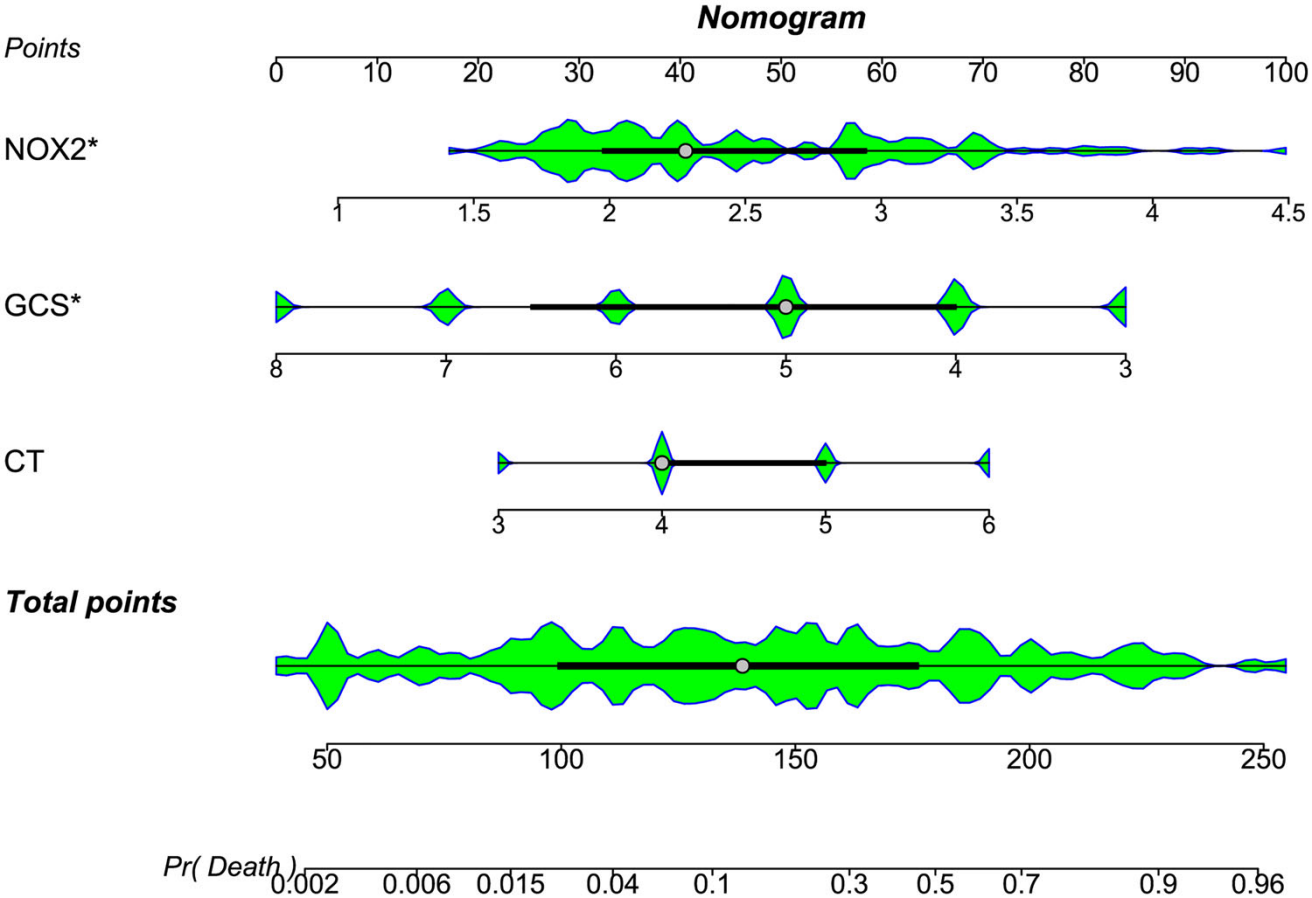
Nomogram assessing death prediction model in individuals with sTBI. Serum NOX2 levels, Rotterdam CT classifications, and GCS scores were combined to differentiate the likelihood of death at 180 days after sTBI. With the help of the nomogram, each variable corresponded to a point, and three points were summed to produce the aggregate scores, which paralleled the designated death risk. NOX2 denotes nicotinamide adenine dinucleotide phosphate oxidase 2; GCS, Glasgow coma scale; CT, computerized tomography; sTBI, severe traumatic brain injury.

**FIGURE 7:**
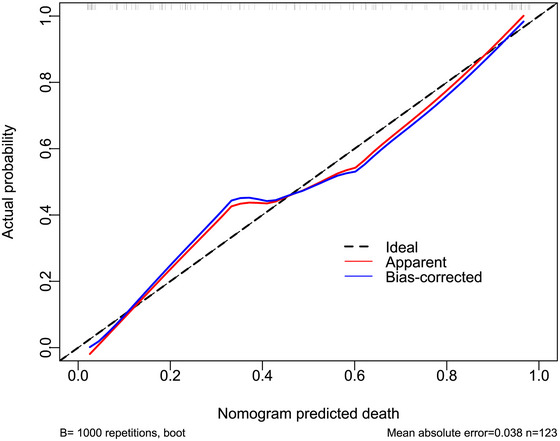
Reliability of nomogram in predicting death at 180 days after sTBI. Calibration plot showed that the constructed framework remained consistent in forecasting death at 180 days following the onset of sTBI, in light of relatively small mean absolute error. sTBI stands for severe traumatic brain injury.

**FIGURE 8:**
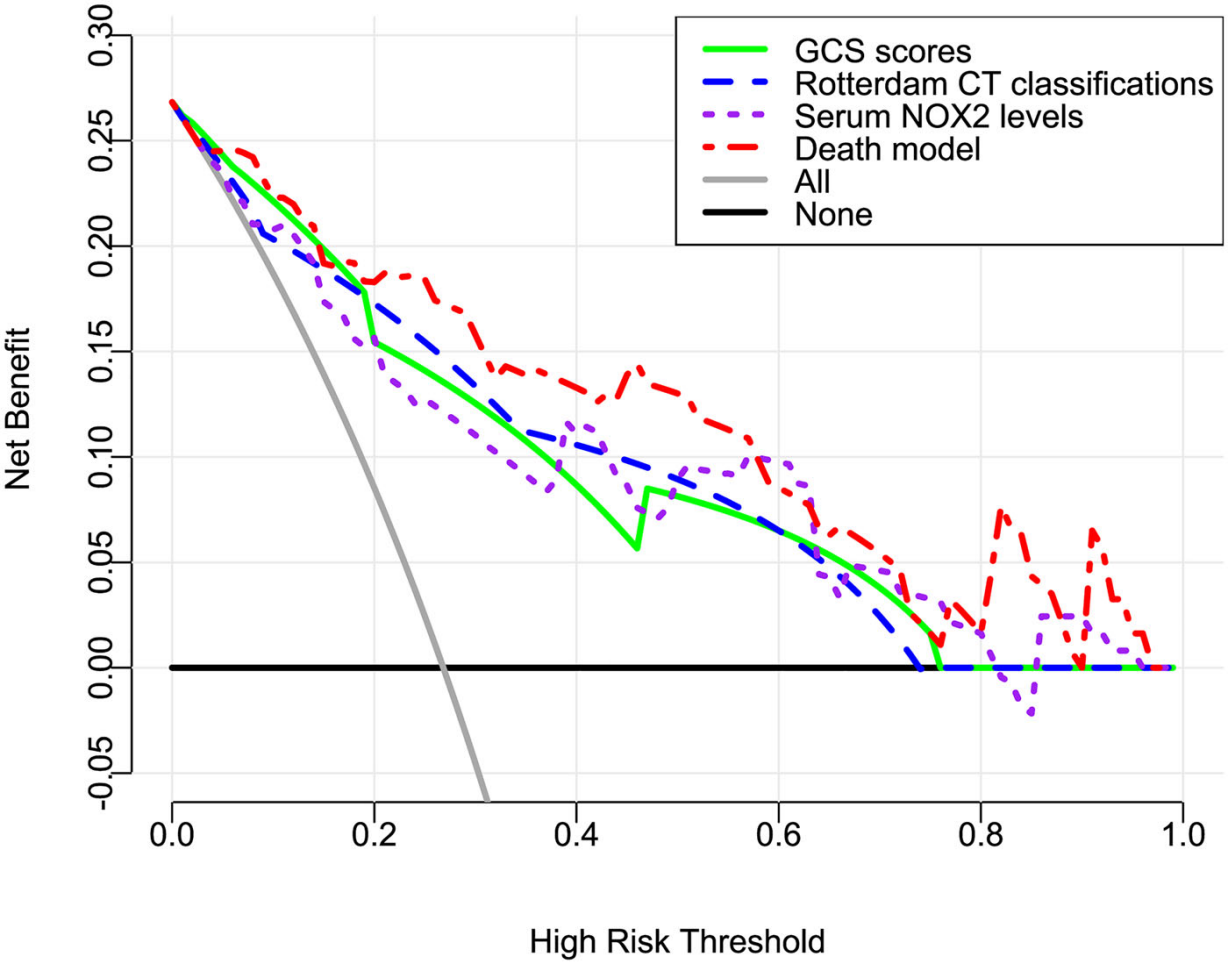
Clinical applicability of the combined model in identifying the likelihood of death at 180 days after sTBI. Based on the background of the decision curve, the developed model demonstrated clinical utility in forecasting mortality at 180 days following sTBI because its curve was relatively far away from the grey line and the black line. NOX2 is indicative of nicotinamide adenine dinucleotide phosphate oxidase 2; GCS, Glasgow coma scale; CT, computerized tomography; sTBI, severe traumatic brain injury.

The AUC for serum NOX2 levels in the ROC curve analysis was greater than 0.750 (Figure 3). In the ROC analysis (Figure 4), the predictive ability of serum NOX2 levels for 180‐day mortality in sTBI patients was similar to those of the GCS scores (AUC, 0.793; 95% CI, 0.711‐0.861; *p* = 0.905) and Rotterdam CT classifications (AUC, 0.799; 95% CI, 0.718‐0.866; *p* = 0.822). Individuals with sTBI showed a linear connection between serum NOX2 levels and the chance of death within 180 days in the restricted cubic spline (P nonlinear > 0.05; Figure 5). In order to predict the death risk, the components that were shown to be strongly connected in the multivariate analysis, that is, serum NOX2 levels, GCS scores, and Rotterdam CT classification, were combined to build a prediction model. And the model was graphically represented by the nomogram (Figure 6). In the nomogram, the total score was summed by adding the values associated with the three criteria, and a greater total score indicated an enhanced likelihood of mortality. The predictive ability of the combined model (AUC, 0.898; 95% CI, 0.830‐0.945) was significantly higher than those of GCS scores (*P* < 0.001), Rotterdam CT classifications (*p* = 0.001), and serum NOX2 levels (*p* = 0.032) (Figure 4). In the calibration curve, the mean absolute error equaled 0.038 (Figure 7), so the model remained consistent in forecasting mortality at 180 days following the onset of sTBI. In the decision curve (Figure 8), the model had a designated curve higher than the gray line and the black line, meaning the developed model demonstrated clinical utility in forecasting mortality at 180 days following sTBI.

### 3.5 Serum NOX2 Levels and Poor 180‐Day Prognosis After sTBI

**FIGURE 9:**
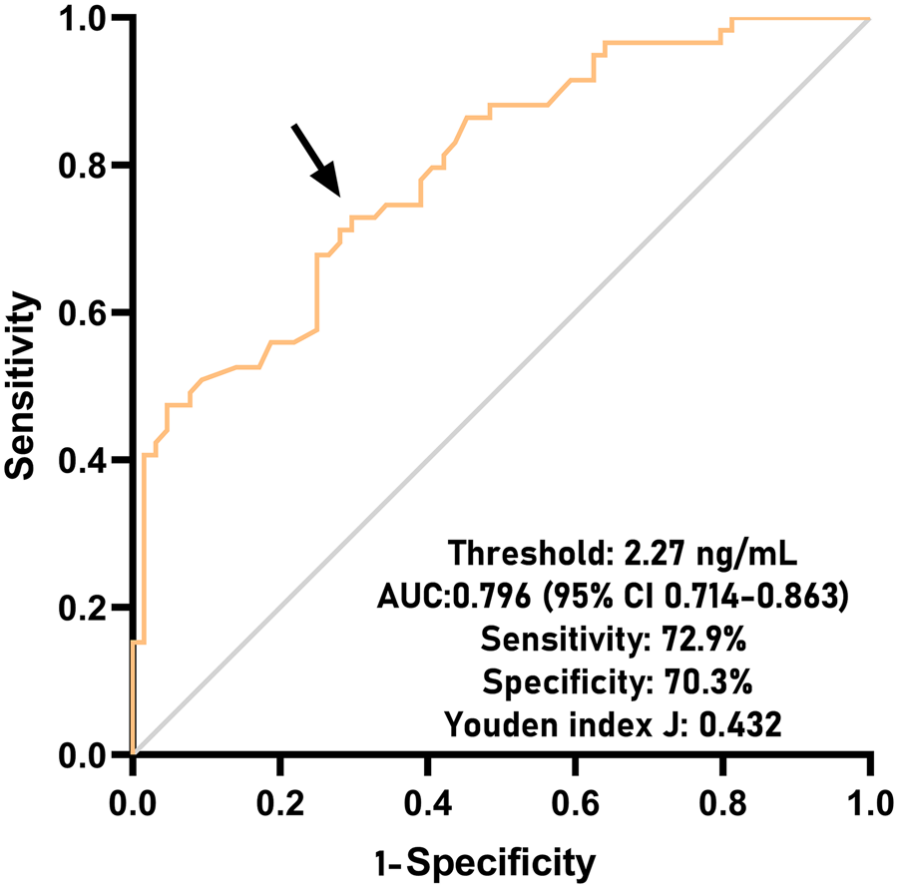
Ability of serum NOX2 levels to predict 180‐day poor prognosis after sTBI. Under the receiver operating characteristic curve, 180‐day poor prognosis was effectively anticipated by serum NOX2 levels, and the Youden approach was adopted to select the criterion of serum NOX2 levels for prognosticating poor prognosis with the maximum Youden value. NOX2 denotes nicotinamide adenine dinucleotide phosphate oxidase 2; sTBI, severe traumatic brain injury; AUC, area under curve; 95% CI, 95% confidence interval.

**FIGURE 10:**
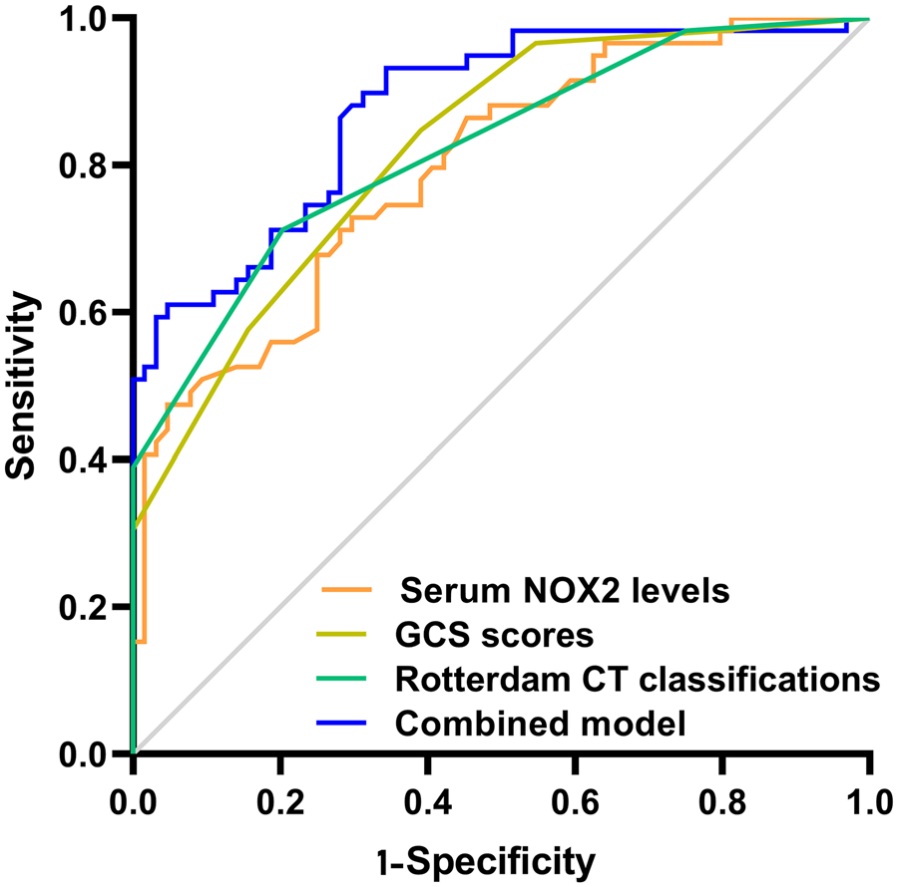
Evaluation of predictive value for post‐sTBI 180‐day poor prognosis between serum NOX2 levels, GCS scores, Rotterdam CT classifications, and the combined model. By aidance of the ROC curve, serum NOX2 levels had predictive ability similar to those of GCS scores and Rotterdam CT classifications (both *P* > 0.05). The preceding three parameters were incorporated to establish a prediction model. The combined model had a significantly higher predictive effect than their independent application (all *P* < 0.05). NOX2 signifies nicotinamide adenine dinucleotide phosphate oxidase 2; GCS, Glasgow coma scale; CT, computerized tomography; sTBI, severe traumatic brain injury; ROC, receiver operating characteristic.

**FIGURE 11:**
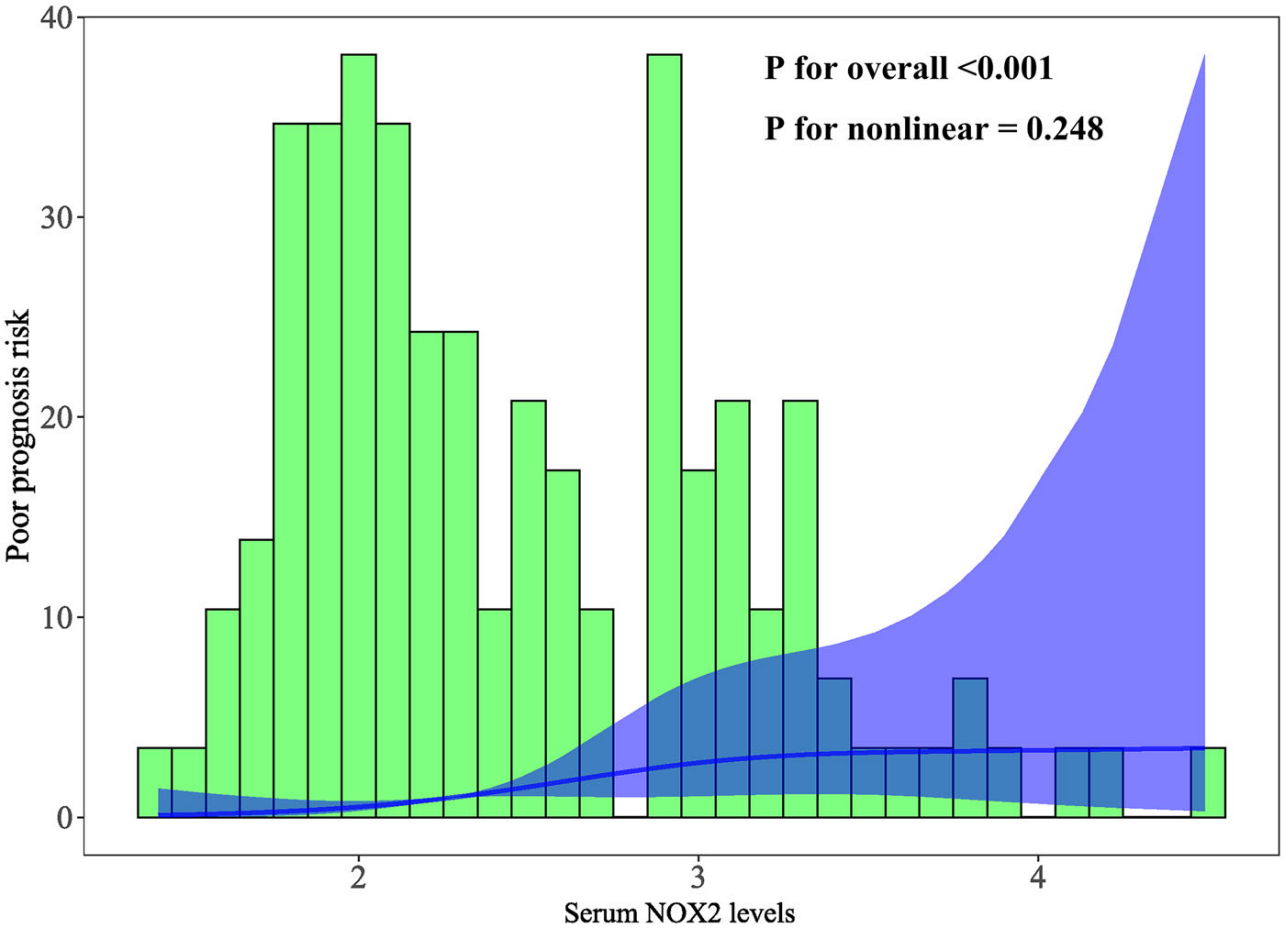
Linear correlation of serum NOX2 levels with the chance of poor prognosis at 180 days following sTBI. In the framework of restricted cubic spline analysis, there was a statistically significant linear correlation between serum NOX2 levels and poor prognosis at 180 days post‐trauma (*p* > 0.05). NOX2 stands for nicotinamide adenine dinucleotide phosphate oxidase 2; sTBI, severe traumatic brain injury.

**FIGURE 12:**
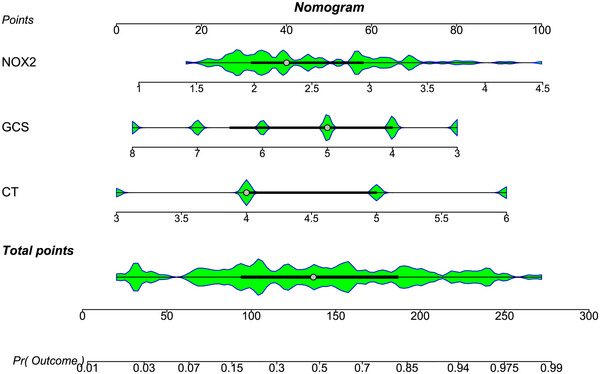
Nomogram assessing the predictive model for 180‐day poor prognosis in individuals with sTBI. Serum NOX2, Rotterdam CT classifications, and GCS scores were combined to differentiate the likelihood of poor prognosis at 180 days after sTBI. In the nomogram, the total scores from three variables could be adopted to predict the risk of poor prognosis at 180 days following sTBI. NOX2 denotes nicotinamide adenine dinucleotide phosphate oxidase 2; GCS, Glasgow coma scale; CT, computerized tomography; sTBI, severe traumatic brain injury.

**FIGURE 13:**
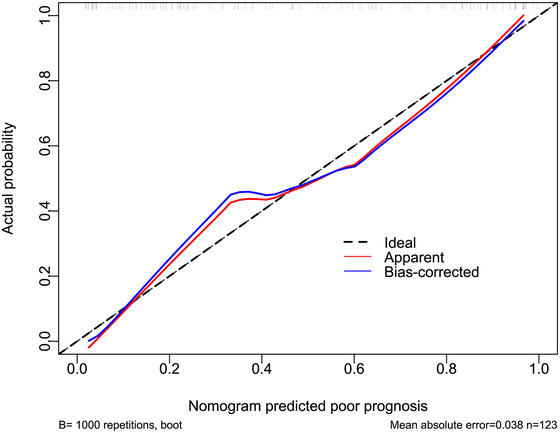
Reliability of the nomogram in predicting the likelihood of poor prognosis at 180 days after sTBI. Following assessment by the calibration curve analysis, the constructed framework had a strong consensus in forecasting poor prognosis at 180 days following the onset of sTBI, according to relatively low absolute mean error. sTBI stands for severe traumatic brain injury.

**FIGURE 14:**
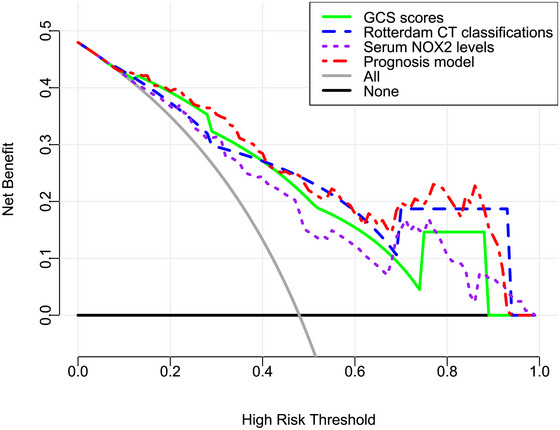
Clinical practicability of the model in identifying the possibility of poor prognosis at 180 days following sTBI. In the paradigm of the decision curve analysis, the developed model exhibited satisfactory clinical usefulness in forecasting poor prognosis at 180 days following sTBI, considering that the model’ curve was relatively far away from the grey line and black line. NOX2 denotes nicotinamide adenine dinucleotide phosphate oxidase 2; GCS, Glasgow coma scale; CT, computerized tomography; sTBI, severe traumatic brain injury.

Based on their GOSE scores, 59 patients had a poor prognosis, and 64 patients showed a good prognosis. Supplemental Figure 9 demonstrates that those with poor prognosis had significantly higher serum NOX2 levels (*P* < 0.001) in comparison to those with good prognosis. The ROC curve analysis confirmed that serum NOX2 levels more than 2.27 ng/mL clearly differentiated poor prognosis patients from those who had a good prognosis at 180 days after trauma (Figure 9). When comparing patients with good and poor prognoses, Table 8 shows significant differences in GCS scores, Rotterdam CT classifications, midline shifts > 5 mm, abnormal cisterns, epidural and subdural hematomas, blood leukocyte counts, blood CRP, glucose, and NOX2 levels (all *P* < 0.05). Table 9 shows similar results verified by univariate logistic regression analysis (all *P* < 0.05). With the inclusion of the preceding significantly distinct variables in the binary logistic regression analysis, GCS scores, Rotterdam CT classifications, and serum NOX2 levels appeared as independently predictive indicators of poor prognosis at 180 days post‐trauma (all *P* < 0.05; Table 9). As shown under the ROC curve (Figure 9), serum NOX2 was an efficient indicator of poor prognosis at 180 days post‐injury, with an AUC of 0.796. Figure 10 shows that serum NOX2 had the AUC similar to those of Rotterdam CT classifications (0.823; 95% CI, 0.744‐0.886; *p* = 0.499) and GCS scores (0.822; 95% CI, 0.743‐0.885; *p* = 0.533), indicating that serum NOX2 may have the potential to predict the poor prognosis after sTBI. In individuals with sTBI, serum NOX2 levels exhibited a significant correlation with the probability of poor prognosis at 180 days when using the restricted cubic spline (P nonlinear > 0.05; Figure 11). The three independent predictors of poor prognosis, namely, GCS scores, serum NOX2 levels, and baseline Rotterdam CT classification, were incorporated to form a prediction model. The model was visually exhibited by applying the nomogram for risk prediction (Figure 12). In the nomogram, the cumulative score was determined by summing the scores for the three specified factors, and a higher score signifies an increased probability of a poor prognosis. As seen in Figure 10, our combined model demonstrated significantly improved predictive capacity (AUC = 0.876; 95% CI, 0.804‐0.928) when contrasted with serum NOX2 levels (*p* = 0.017), GCS scores (*p* = 0.006), and Rotterdam CT classifications (*p* = 0.008). The calibration curve evaluated the reliability of the model in predicting poor prognosis at 180 days after sTBI. Figure 13 shows that the mean absolute error was 0.038, indicating the model had medium to high stability to predict poor prognosis after sTBI. The decision curve assessed the clinical applicability of the model in identifying the likelihood of poor prognosis at 180 days after trauma. The curve owned by the model is higher than the gray line and the black line (Figure 14), signifying that the model had clinical utility in forecasting poor prognosis at 180 days following sTBI.

**TABLE 8:** Factors associated with 180‐day function prognosis in severe traumatic brain injury

	Good prognosis (n=64)	Poor prognosis (n=59)	*Z/χ^2^*	*P* value
Gender (male/female)	28/36	29/30	0.360	0.548
Age (years)	49 (35‐61)	53 (34‐61)	−0.403	0.687
Current cigarette smoking	16 (25.0%)	16 (27.1%)	0.072	0.789
Alcohol abuse	18 (28.1%)	21 (35.6%)	0.791	0.374
Hypertension	14 (21.9%)	18 (30.5%)	1.189	0.276
Diabetes mellitus	8 (12.5%)	10 (16.9%)	0.486	0.486
Hyperlipidemia	17 (26.6%)	18 (30.5%)	0.235	0.628
Hospital admission time (h)	4.3 (2.9‐5.9)	4.1 (2.7‐5.6)	−0.679	0.497
Blood‐sampling time (h)	5.5 (3.6‐6.8)	5.1 (3.7‐6.8)	−0.425	0.671
Systolic arterial pressure (mmHg)	117 (105‐132)	110 (91‐123)	−1.699	0.089
Diastolic arterial pressure (mmHg)	74 (66‐82)	71 (54‐84)	−1.155	0.248
Traumatic causes			4.049	0.132
Automobile/motorcycle	36 (56.3%)	29 (49.2%)	—	—
Fall/jump	23 (35.9%)	18 (30.5%)	—	—
Others	5 (7.8%)	12 (20.3%)	—	—
GCS scores	6 (5‐7)	4 (3‐5)	−6.275	<0.001
Rotterdam CT classification	4 (3‐4)	5 (4‐6)	−6.506	<0.001
Midline shift > 5 mm	29 (45.3%)	42 (71.2%)	8.422	0.004
Abnormal cisterns	40 (62.5%)	51 (86.4%)	9.142	0.002
Epidural hematoma	28 (43.8%)	39 (66.1%)	6.184	0.013
Subdural hematoma	30 (46.9%)	39 (66.1%)	4.608	0.032
Subarachnoid hemorrhage	38 (59.4%)	44 (74.6%)	3.192	0.074
Intraventricular hemorrhage	5 (7.8%)	13 (22.0%)	4.970	0.026
Intracerebral hematoma	32 (50.0%)	38 (64.4%)	2.598	0.107
Brain contusion	39 (60.9%)	48 (81.4%)	6.182	0.013
Pneumocephalus	26 (40.6%)	21 (35.6%)	0.329	0.566
Blood leukocyte count (×10^9^/L)	8.5 (6.4‐9.8)	9.6 (7.5‐11.3)	−2.476	0.013
Blood CRP levels (mg/L)	3.1 (1.8‐8.8)	8.0 (3.1‐13.0)	−2.987	0.003
Blood potassium levels (mmol/L)	3.63 (3.43‐3.97)	3.66 (3.39‐3.89)	−0.296	0.767
Blood glucose levels (mmol/l)	6.2 (5.1‐9.2)	9.5 (6.8‐14.2)	−3.876	<0.001
Serum NOX2 levels (ng/mL)	2.05 (1.84‐2.53)	2.89 (2.23‐3.34)	−5.663	<0.001

*Note*: Qualitative variables were presented as counts (percentages) and were compared for intergroup difference using the chi‐square test or Fisher's exact test, as appropriate. Quantitative variables were summarized as medians (upper and lower quartiles) or as means ± standard deviations, as appropriate. Intergroup comparisons were done using an unpaired Student's t‐test or Mann‐Whitney U test where appropriate.

Abbreviations: CRP, C‐reactive protein; CT, computerized tomography; GCS, Glasgow coma scale; NOX2, nicotinamide adenine dinucleotide phosphate oxidase 2.

**TABLE 9:** Univariate and multivariate logistic regression analysis of predictors of 180‐day poor prognosis after severe traumatic brain injury

	Univariate analysis		Multivariate analysis	
	Odds ratio (95% *CI*)	*P* value	Odds ratio (95% *CI*)	*P* value
Gender (male/female)	0.805 (0.395‐1.637)	0.549	—	—
Age (y)	1.005 (0.982‐1.029)	0.660	—	—
Current cigarette smoking	1.116 (0.499‐2.499)	0.789	—	—
Alcohol abuse	1.412 (0.659‐3.026)	0.375	—	—
Hypertension	1.568 (0.697‐3.529)	0.277	—	—
Diabetes mellitus	1.429 (0.523‐3.905)	0.487	—	—
Hyperlipidemia	1.214 (0.554‐2.659)	0.628	—	—
Hospital admission time (h)	0.934 (0.799‐1.091)	0.387	—	—
Blood‐sampling time (h)	0.961 (0.827‐1.116)	0.602	—	—
Systolic arterial pressure (mmHg)	0.990 (0.976‐1.005)	0.181	—	—
Diastolic arterial pressure (mmHg)	0.988 (0.965‐1.010)	0.285	—	—
Traumatic causes		0.151	—	—
Automobile/motorcycle	1		—	—
Fall/jump	0.336 (0.106‐1.062)	0.063	—	—
Others	0.326 (0.097‐1.096)	0.070	—	—
GCS scores	0.369 (0.257‐0.530)	<0.001	0.438 (0.224‐0.855)	0.016
Rotterdam CT classification	5.867 (3.136‐10.976)	<0.001	3.321 (1.200‐10.124)	0.022
Midline shift > 5 mm	2.982 (1.441‐6.300)	0.004	2.180 (0.577‐8.234)	0.250
Abnormal cisterns	3.825 (1.554‐9.416)	0.004	2.159 (0.479‐9.737)	0.317
Epidural hematoma	2.507 (1.207‐5.208)	0.014	1.059 (0.264‐4.247)	0.935
Subdural hematoma	2.210 (1.066‐4.582)	0.033	1.162 (0.328‐4.116)	0.817
Subarachnoid hemorrhage	2.007 (0.930‐4.333)	0.076	—	—
Intraventricular hemorrhage	3.335 (1.109‐10.029)	0.032	1.126 (0.160‐7.929)	0.905
Intracerebral hematoma	1.906 (0.927‐3.919)	0.079	—	—
Brain contusion	2.797 (1.225‐6.386)	0.015	2.025 (0.450‐9.117)	0.358
Pneumocephalus	0.808 (0.389‐1.676)	0.566	—	—
Blood leukocyte count (×10^9^/L)	1.227 (1.045‐1.442)	0.013	1.010 (0.775‐1.316)	0.943
Blood CRP levels (mg/L)	1.061 (1.002‐1.123)	0.044	1.044 (0.946‐1.152)	0.389
Blood potassium levels (mmol/L)	1.044 (0.496‐2.196)	0.910	—	—
Blood glucose levels (mmol/l)	1.202 (1.082‐1.334)	0.001	1.170 (0.977‐1.400)	0.088
Serum NOX2 levels (ng/mL)	7.523 (3.396‐16.668)	<0.001	4.841 (1.262‐18.567)	0.021

*Note*: Results were presented as odds ratios (95% confidence interval) using the univariate and multivariate logistic regression analysis. 95% CI indicates 95% confidence interval.

Abbreviations: CRP, C‐reactive protein; CT, computerized tomography; GCS, Glasgow coma scale; NOX2, nicotinamide adenine dinucleotide phosphate oxidase 2.

## 4 Discussion

The early identification of outcomes in sTBI patients is essential for directing therapeutic care. This single‐center, prospective cohort research evaluated the prognostic significance of serum NOX2 levels regarding survival outcomes and long‐term neurological prognosis in patients with sTBI. Four key findings emerged from our investigation. At first, when symptoms of sTBI were first developing, serum NOX2 levels were significantly elevated. Second, there was a correlation between the Rotterdam CT classifications and GCS scores, as well as serum NOX2 levels upon admission. Third, there was a fairly strong selective potential to predict death and poor 180‐day prognosis by independently correlating serum NOX2 levels with both mortality and poor prognosis. Fourth, we observed a significant difference between the AUC of the combined model (serum NOX2 levels, GCS scores, and Rotterdam CT classifications) and that of each individual variable, with the combined model significantly improving prognostic predictive ability. Thus, it comes to conclusion that serum NOX2 may be a promising prognostic indicator of sTBI.

Extensive research has been conducted in the last several decades on the processes that cause subsequent brain damage after an acute brain injury (Lazaridis et al. 2019, Khatri et al. 2021). Oxidative stress is strongly linked to neurological outcomes in individuals with acute brain injuries and has been shown to have a critical role in subsequent brain damage in several studies (Shao et al. 2022, Wang et al. 2018). Most organisms rely on NADPH, which is essential for cellular functioning and hydrogen transport in chemical processes (Ying 2008). NOX is an essential protein that plays a role in the cell's oxidative functional activity (Pecchillo Cimmino et al. 2023). NOX2, one of several protein isoforms in the NOX family, is mostly found in the cytoplasm and on the plasma membrane; it is involved in oxidative stress activation and increases superoxide production (Vermot et al. 2021). Hippocampal neurons, microglia, astrocytes, and cerebral endothelial cells are the main sites of NOX2 expression (Fan et al. 2019). Neurons and glial cells in particular exhibit dramatically increased NOX2 expression in rat brain tissues after craniocerebral trauma (Kumar et al. 2016). Acute cerebral ischemia and aSAH models have also shown similar results (Wang et al. 2020, Miao et al. 2023). Likewise, serum NOX2 levels were significantly elevated in this cohort of sTBI patients. Our results are strongly supportive of the notion that serum NOX2 may be potentially originated from damaged brain tissues. Nonetheless, NOX2 is also produced by peripheral immune cells (Vermot et al. 2021), and hence it remains unclear whether elevated serum NOX2 specifically reflects brain injury or systemic inflammation. For the sake of investigating possible sources of serum NOX2, it is imperative that NOX2 protein in cerebrospinal fluid and mRNA in peripheral blood cells be determined in the future so as to complete a comprehensive analysis.

As for NOX2 functions, rats subjected to SAH have shown improved neurological function, less neuronal death and degeneration caused by SAH, and less ROS production in brain tissue when their NOX2 expression was specifically inhibited (Miao et al. 2023). Reducing infarct extent, attenuating brain tissue damage, and delaying the course of neuronal loss following stroke were all effects of NOX2 knockdown in mice with acute cerebral ischemia (McCann et al. 2014). Brain hemorrhage models showed that NOX2 deletion mice had a much lower hematoma volume, neurological disability, and death rate (Gao et al. 2021). In a rat neuron‐microglia co‐culture paradigm, oxidative stress and inflammatory responses were markedly reduced when NOX2 was knocked down using siRNA (Zeng et al. 2018). In addition, an in vitro BBB model confirmed the protective impact of NOX2‐specific inhibition on BBB function and integrity (Kadir et al. 2021). Also, there was an inhibition of oxyhemoglobin‐induced neuronal apoptosis in experimental SAH when NOX2 was silenced using siRNA (Zhang et al. 2017). Thus, NOX2 may be a promising therapeutic target of acute brain injury (Ma et al. 2018). The expression of NOX2 in neurons and astrocytes of TBI patients reached its highest point 12–24 h after the injury and was highly associated with the severity of the condition (Li et al. 2015). Researchers have discovered that individuals with brain damage illnesses, such as neurodegenerative diseases and aSAH, have considerably higher serum NOX2 levels. These levels are directly linked to the severity of the condition (Wu et al. 2023, Loffredo et al. 2020). In particular, the WFNS classification, the Hunt‐Hess classification, and the mFisher scores upon admission were positively and substantially linked with serum NOX2 levels among aSAH patients within the early stages of disease (Wu et al. 2023). A modified Rankin Scale score at 90 days after stroke showed a high correlation with these values as well (Wu et al. 2023). Patients with delayed cerebral ischemia and a poor outcome were more likely to have elevated serum NOX2 levels (Wu et al. 2023). In this prospective cohort study, serum NOX2 levels of 123 patients with sTBI were independently associated with patients' GCS score and Rotterdam CT classification and, meanwhile, effectively differentiated the risk of death and poor prognosis (GOSE scores). Notably, the predictive power of serum NOX2 levels was comparable to that of the GCS score and Rotterdam CT classifications. Interestingly, the combined model of serum NOX2 levels, GCS scores, and Rotterdam CT classifications demonstrated significantly greater predictive ability for death and poor prognosis at 180 days post‐injury, as compared to the individual use of Rotterdam CT classifications, GCS scores, or serum NOX2 levels. In conclusion, serum NOX2 levels may serve as a potential biomarker for assessing disease severity and predicting death and poor prognosis following sTBI. Although our study did not explore causal effects, NOX2, clearly acting as a detrimental factor, activates oxidative stress, with extensive involvement in the process of secondary brain injury after acute brain injury, including TBI (Ma et al. 2018), therefore leading to the conception that NOX2‐mediated oxidative stress may contribute to worse outcomes after sTBI.

As far as we are aware, this may be the first series to confirm markedly elevated serum NOX2 levels after sTBI and its potential prognostic role of clinical value. Our results are supported by multivariate analysis, nomogram models, calibration curves, and decision curves, therefore showing that it may be more reliable and scientifically valid to use serum NOX2 levels to predict mortality and long‐term neurological outcomes in sTBI patients. However, there are still several weaknesses here. First, the GOSE is often applied to evaluate the long‐term prognosis of patients with sTBI. However, the GOSE is not a very sensitive measure of long‐term outcome and also comes with its own limitations. Therefore, a stronger battery of cognitive and adaptive functioning measures should be used for assessing functional outcome of sTBI in the future. Second, the GCS may not be the best indicator of injury severity as compared to other indicators of recovery rate, such as duration of coma, time to follow commands, and length of posttraumatic amnesia/confusional state; and thus, it might be a good choice to supplement such indicators of injury severity in future studies. Third, only one measurement of serum NOX2 levels was taken within 12 h of sTBI in this study. The exploration of biomarker dynamics is important after sTBI, so serial measurements, for example, at hours 12, 24, and 72 h, would better define the evolutional trajectory of serum NOX2 levels and further determine their clinical values. Hence, it may be a future research direction that serum NOX2 levels would be detected at multiple time points following sTBI, wherein its longitudinal change is discovered so that some valuable information can be proffered. Last, although the sample population is sufficient according to statistical sample size calculation in this study, the study is limited to a single hospital without external validation, therefore necessitating further multicenter, prospective validation studies in order to facilitate the universality and generalization of the study conclusions.

## 5 Conclusion

Elevated serum NOX2 levels of patients with sTBI are closely linked to higher Rotterdam CT classifications and lower GCS scores. Additionally, serum NOX2 levels are independently associated with mortality and poor prognosis 180 days post‐TBI. Also, serum NOX2 levels effectively predict worse clinical outcomes following sTBI. So, it is suggested that serum NOX2 may serve as a prognostic biomarker of sTBI.

## Author Contributions

**Chang Su**: writing – original draft, funding acquisition, data curation, conceptualization, methodology, investigation. **Dapu Shen**: data curation, project administration, writing – review and editing, conceptualization, methodology. **Junlong Xu**: project administration, methodology, conceptualization, formal analysis. **Miaomiao Chen**: project administration, conceptualization, resources. **Heng He**: project administration, software, conceptualization. **Jianping Ye**: writing – review and editing, conceptualization, methodology, validation, supervision, visualization.

## Ethics Statement

The study's methodology was approved by the Lishui People's Hospital Institutional Review Committee (Medical Ethics Review No. 2020‐001, 2020‐002), which ensured compliance with the Declaration of Helsinki standards. Written informed consent forms were acquired from legal representatives of patients and the controls themselves.

## Conflicts of Interest

The authors declare no conflicts of interest.

## Peer Review

The peer review history for this article is available at https://publons.com/publon/10.1002/brb3.70692

## Supporting information




**Supporting Fig.1** Flowchart for participant recruitment. After initially screening 152 participants, 29 were excluded based on the set criteria, leaving 123 patients to be enrolled in the research. sTBI stands for severe traumatic brain injury.

**Supporting fig.2** Boxplot portraying serum NOX2 levels across GCS scores following sTBI. Serum NOX2 levels were substantially decreased in order of post‐sTBI GCS scores from 3 to 8 (*P*<0.001). NOX2 denotes nicotinamide adenine dinucleotide phosphate oxidase 2; GCS, Glasgow coma scale; and sTBI, severe traumatic brain injury.

**Supporting fig.3** Correlogram illustrating the relationship between serum NOX2 levels and GCS scores after sTBI. Serum NOX2 levels were substantially inversely correlated with GCS scores post‐sTBI (*P* < 0.001). NOX2 means nicotinamide adenine dinucleotide phosphate oxidase 2; GCS, Glasgow coma scale; sTBI, severe traumatic brain injury.

**Supporting fig.4** Boxplot delineating serum NOX2 levels among subgroups based on Rotterdam CT classifications after sTBI. Serum NOX2 levels were substantially lowest in patients with the Rotterdam CT score 3, followed by the scores 4 and 5, and were significantly highest in those with the score 6 (*P* < 0.001). NOX2 means nicotinamide adenine dinucleotide phosphate oxidase 2; CT, computed tomography; sTBI, severe traumatic brain injury.

**Supporting fig.5** Correlogram outlining the relationship between serum NOX2 levels and Rotterdam CT classifications subsequent to sTBI. Serum NOX2 levels were in substantially positive proportion to Rotterdam CT scores post‐sTBI (*P* < 0.001). NOX2 means nicotinamide adenine dinucleotide phosphate oxidase 2; CT, computerized tomography; and sTBI, severe traumatic brain injury.

**Supporting fig.6** Boxplot showing serum NOX2 levels among patients with different 180‐day GOSE scores after sTBI. Patients with GOSE score 1 had markedly higher serum NOX2 levels, with a gradual decline in the NOX2 levels in order of GOSE scores from 2 to 7, and those with the score 8 exhibited the notably lowest levels (*P* < 0.001). NOX2 denotes nicotinamide adenine dinucleotide phosphate oxidase 2; GOSE, Glasgow Outcome Scale Extended; sTBI, severe traumatic brain injury.

**Supporting fig.7** Correlogram depicting the relationship between serum NOX2 levels and 180‐day GOSE scores after sTBI. Serum NOX2 levels of patients were highly related to GOSE scores at the 180‐day mark following sTBI (*P* < 0.001). NOX2 indicates serum nicotinamide adenine dinucleotide phosphate oxidase 2; GOSE, Glasgow Outcome Scale Extended; stbi, severe traumatic brain injury.

**Supporting fig.8** Serum NOX2 levels between the alive and the deceased at the 180‐day mark following sTBI. The boxplot showed that serum NOX2 levels of non‐survivors were substantially higher than those of survivors at 180 days post‐sTBI (*P* < 0.001). NOX2 signifies nicotinamide adenine dinucleotide phosphate oxidase 2; sTBI, severe traumatic brain injury.

**Supporting fig.9** Boxplot displaying serum NOX2 levels between patients with good prognosis and those presenting with poor prognosis at 180 days after sTBI. Serum NOX2 levels were significantly higher in subjects with poor prognosis than those with good prognosis at 180 days after sTBI (*P* < 0.001). NOX2 stands for nicotinamide adenine dinucleotide phosphate oxidase 2; sTBI, severe traumatic brain injury.

## Data Availability

The data that support the findings of this study are available on request from the corresponding author. The data are not publicly available due to privacy or ethical restrictions.
