# Supplementary figures and images for: Application of Serum NADPH Oxidase 2 Levels for Predicting 180‐Day Clinical Outcomes Following Severe Traumatic Brain Injury: A Prospective Cohort Analysis
Source: Brain Behav. 2025 Jul 11;15(7):e70692. doi: 10.1002/brb3.70692 (PMC12246553; doi:10.1002/brb3.70692)

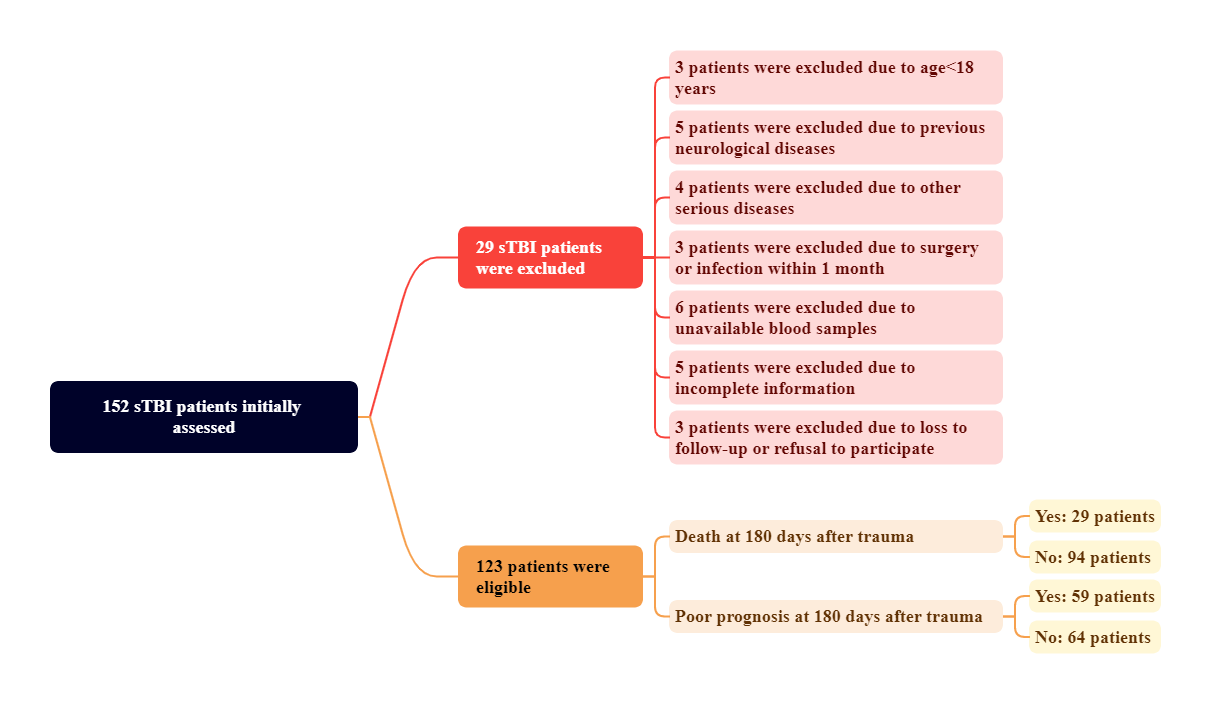

Supplement: Supplementary file 1 — Supporting Fig.1 Flowchart for participant recruitment. After initially screening 152 participants, 29 were excluded based on the set criteria, leaving 123 patients to be enrolled in the research. sTBI stands for severe traumatic brain injury. [file BRB3-15-e70692-s009.tif]

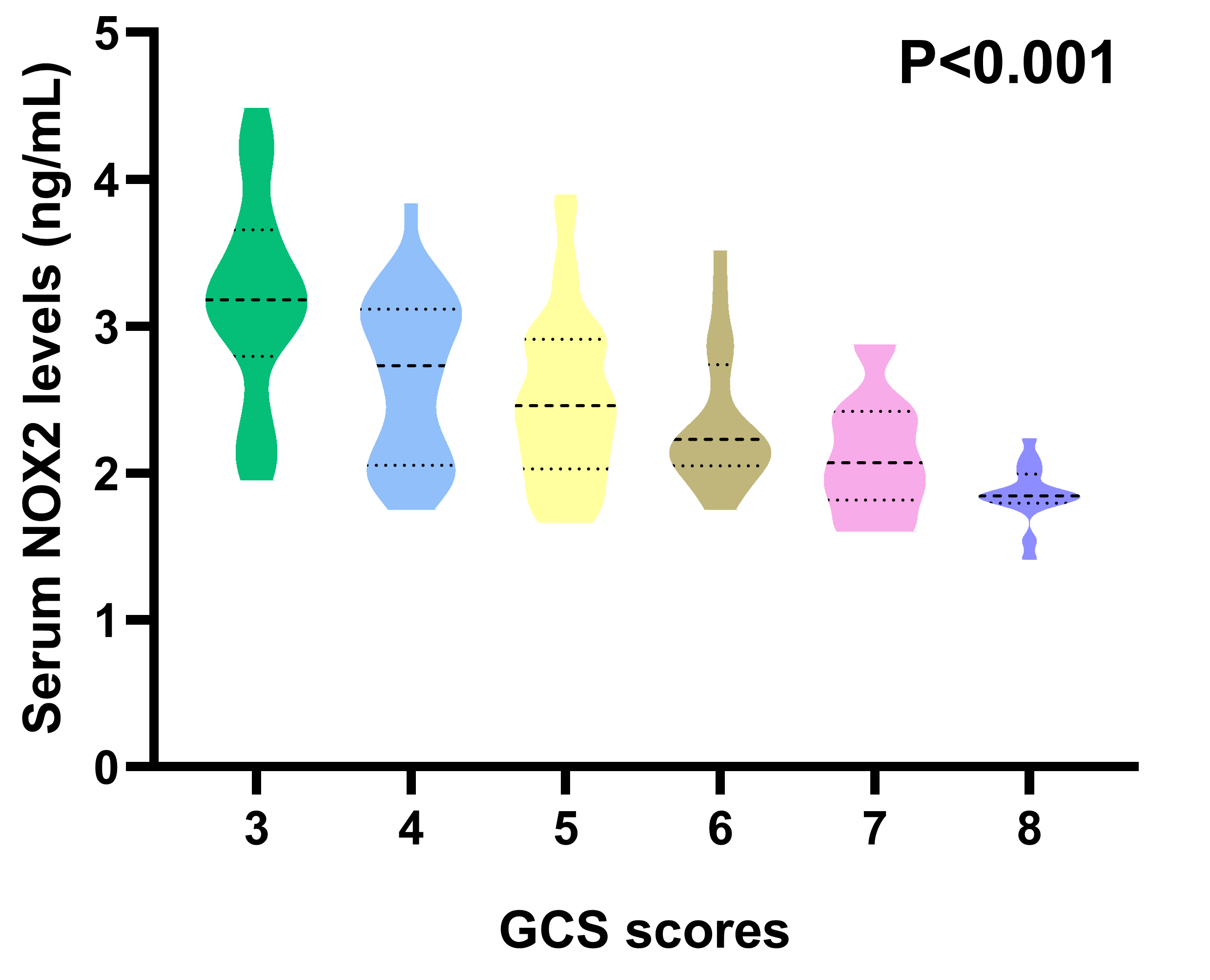

Supplement: Supplementary file 2 — Supporting fig.2 Boxplot portraying serum NOX2 levels across GCS scores following sTBI. Serum NOX2 levels were substantially decreased in order of post‐sTBI GCS scores from 3 to 8 (P<0.001). NOX2 denotes nicotinamide adenine dinucleotide phosphate oxidase 2; GCS, Glasgow coma scale; and sTBI, severe traumatic brain injury. [file BRB3-15-e70692-s008.tif]

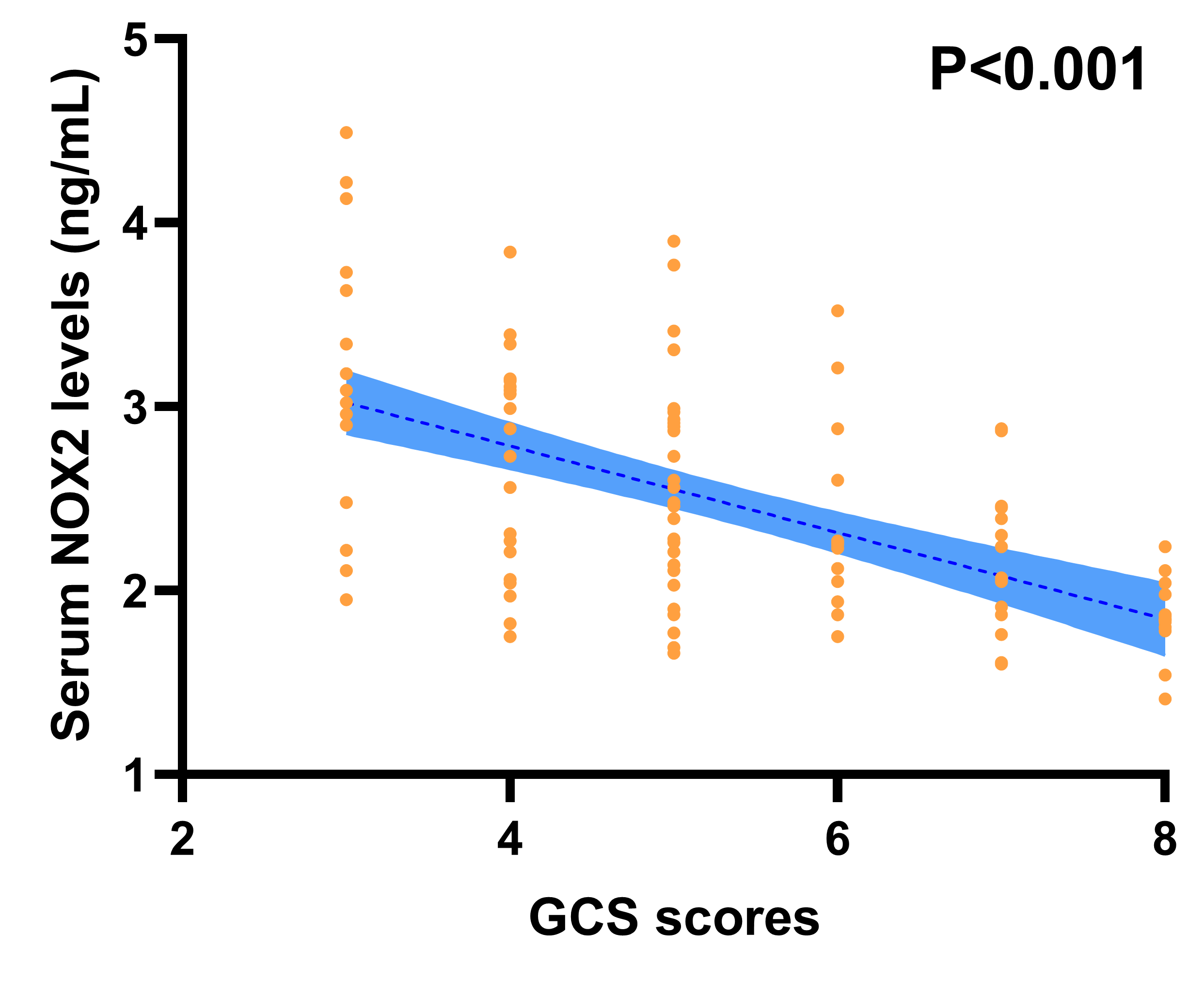

Supplement: Supplementary file 3 — Supporting fig.3 Correlogram illustrating the relationship between serum NOX2 levels and GCS scores after sTBI. Serum NOX2 levels were substantially inversely correlated with GCS scores post‐sTBI (P < 0.001). NOX2 means nicotinamide adenine dinucleotide phosphate oxidase 2; GCS, Glasgow coma scale; sTBI, severe traumatic brain injury. [file BRB3-15-e70692-s003.tif]

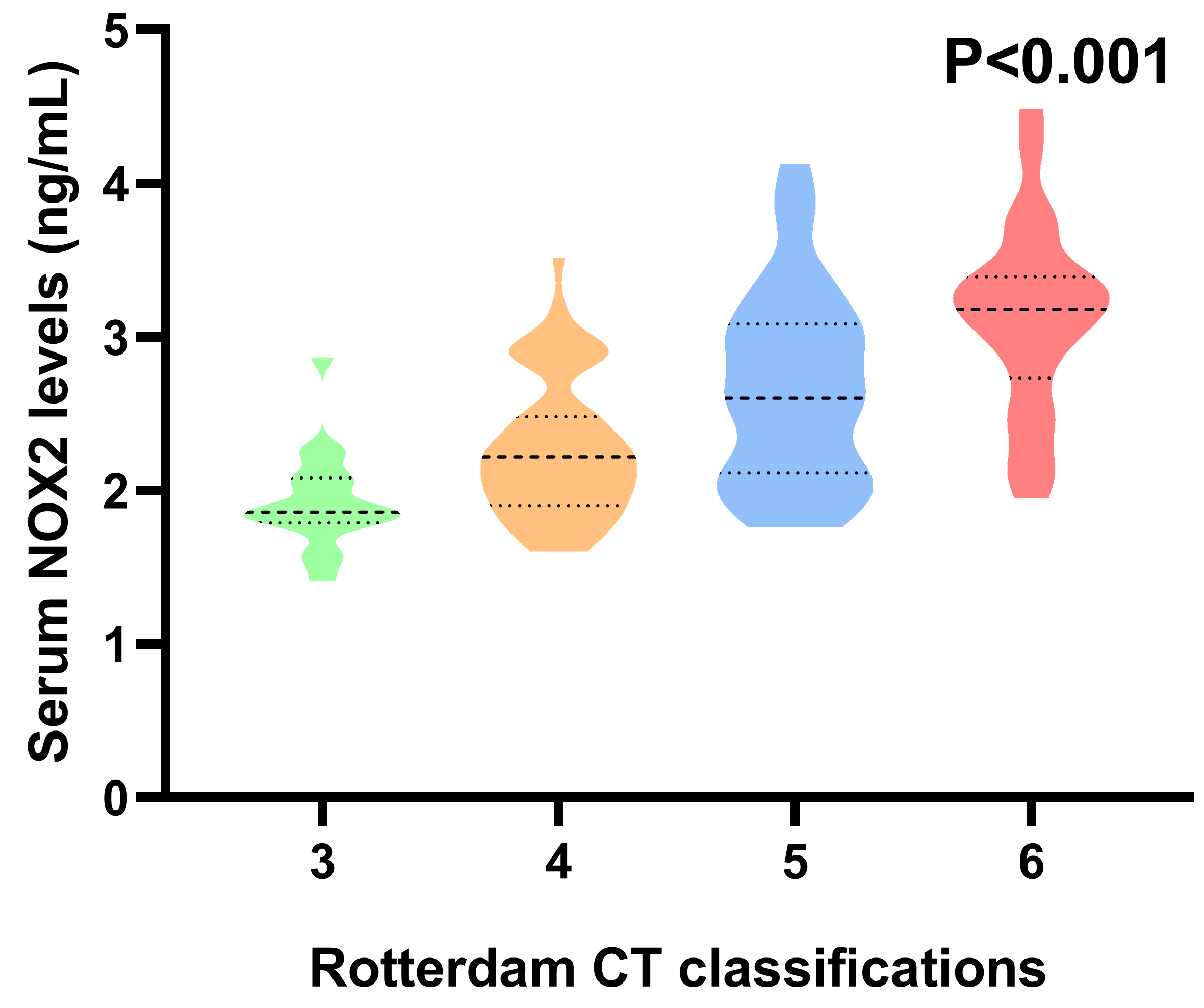

Supplement: Supplementary file 4 — Supporting fig.4 Boxplot delineating serum NOX2 levels among subgroups based on Rotterdam CT classifications after sTBI. Serum NOX2 levels were substantially lowest in patients with the Rotterdam CT score 3, followed by the scores 4 and 5, and were significantly highest in those with the score 6 (P < 0.001). NOX2 means nicotinamide adenine dinucleotide phosphate oxidase 2; CT, computed tomography; sTBI, severe traumatic brain injury. [file BRB3-15-e70692-s001.tif]

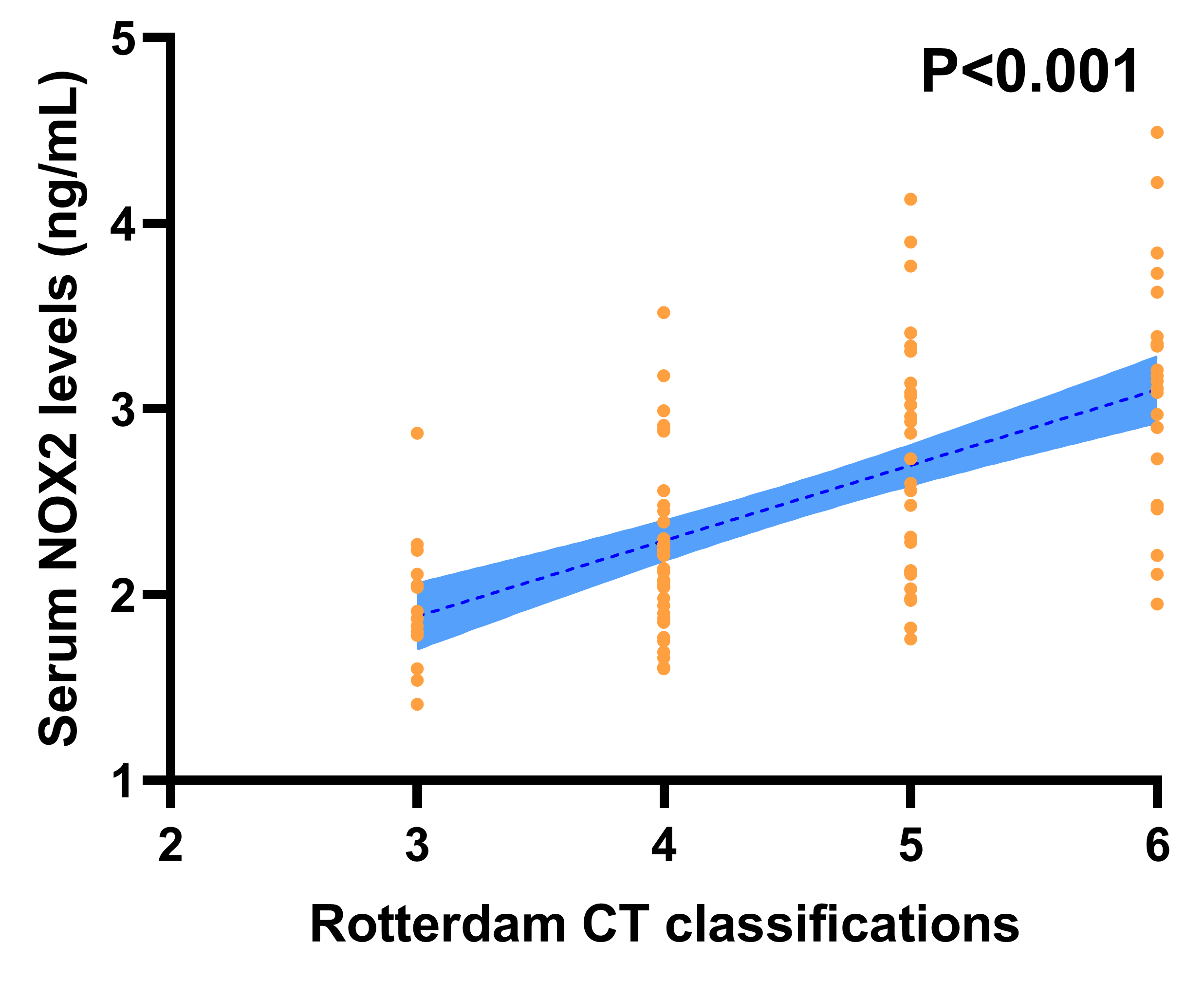

Supplement: Supplementary file 5 — Supporting fig.5 Correlogram outlining the relationship between serum NOX2 levels and Rotterdam CT classifications subsequent to sTBI. Serum NOX2 levels were in substantially positive proportion to Rotterdam CT scores post‐sTBI (P < 0.001). NOX2 means nicotinamide adenine dinucleotide phosphate oxidase 2; CT, computerized tomography; and sTBI, severe traumatic brain injury. [file BRB3-15-e70692-s006.tif]

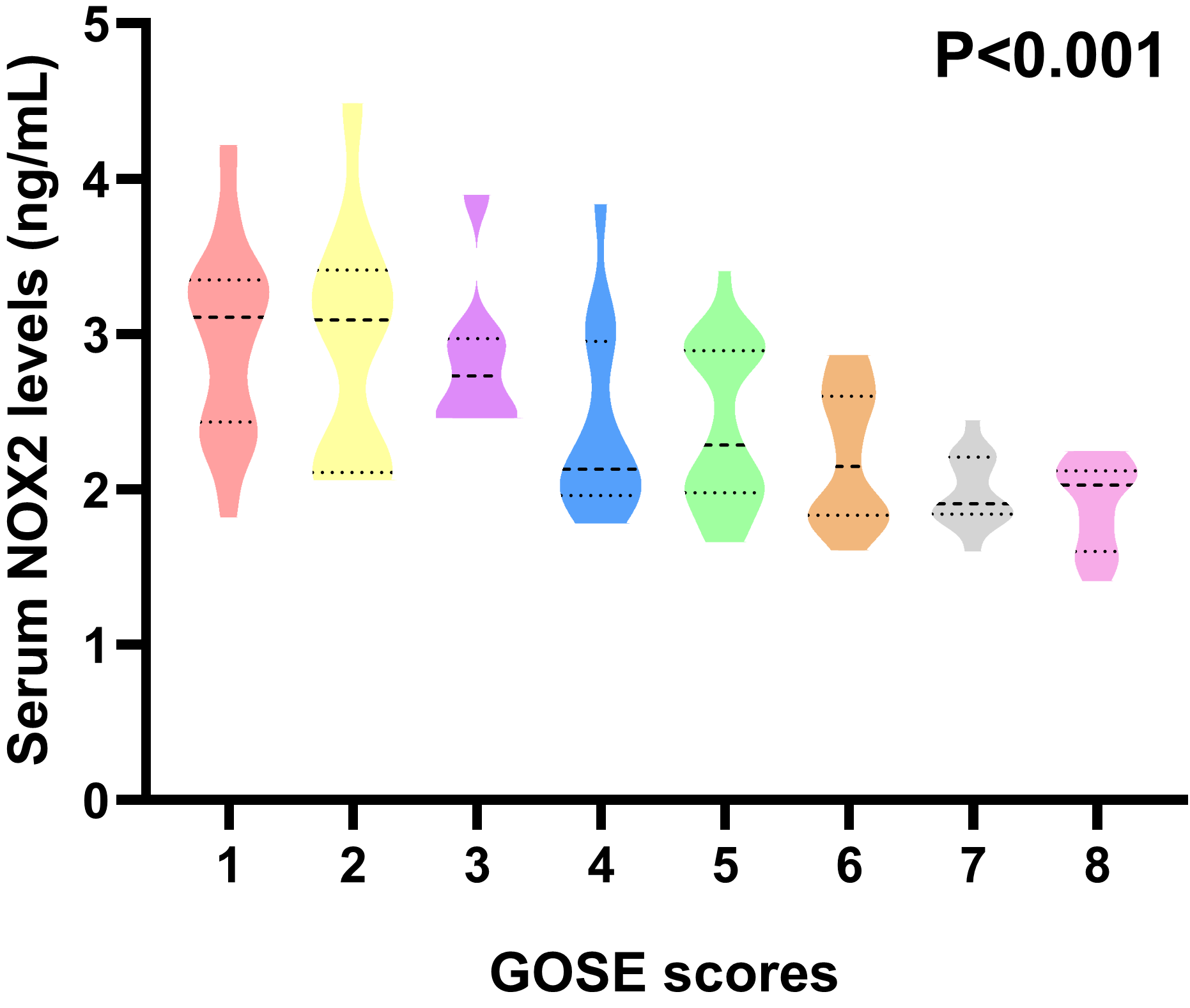

Supplement: Supplementary file 6 — Supporting fig.6 Boxplot showing serum NOX2 levels among patients with different 180‐day GOSE scores after sTBI. Patients with GOSE score 1 had markedly higher serum NOX2 levels, with a gradual decline in the NOX2 levels in order of GOSE scores from 2 to 7, and those with the score 8 exhibited the notably lowest levels (P < 0.001). NOX2 denotes nicotinamide adenine dinucleotide phosphate oxidase 2; GOSE, Glasgow Outcome Scale Extended; sTBI, severe traumatic brain injury. [file BRB3-15-e70692-s005.tif]

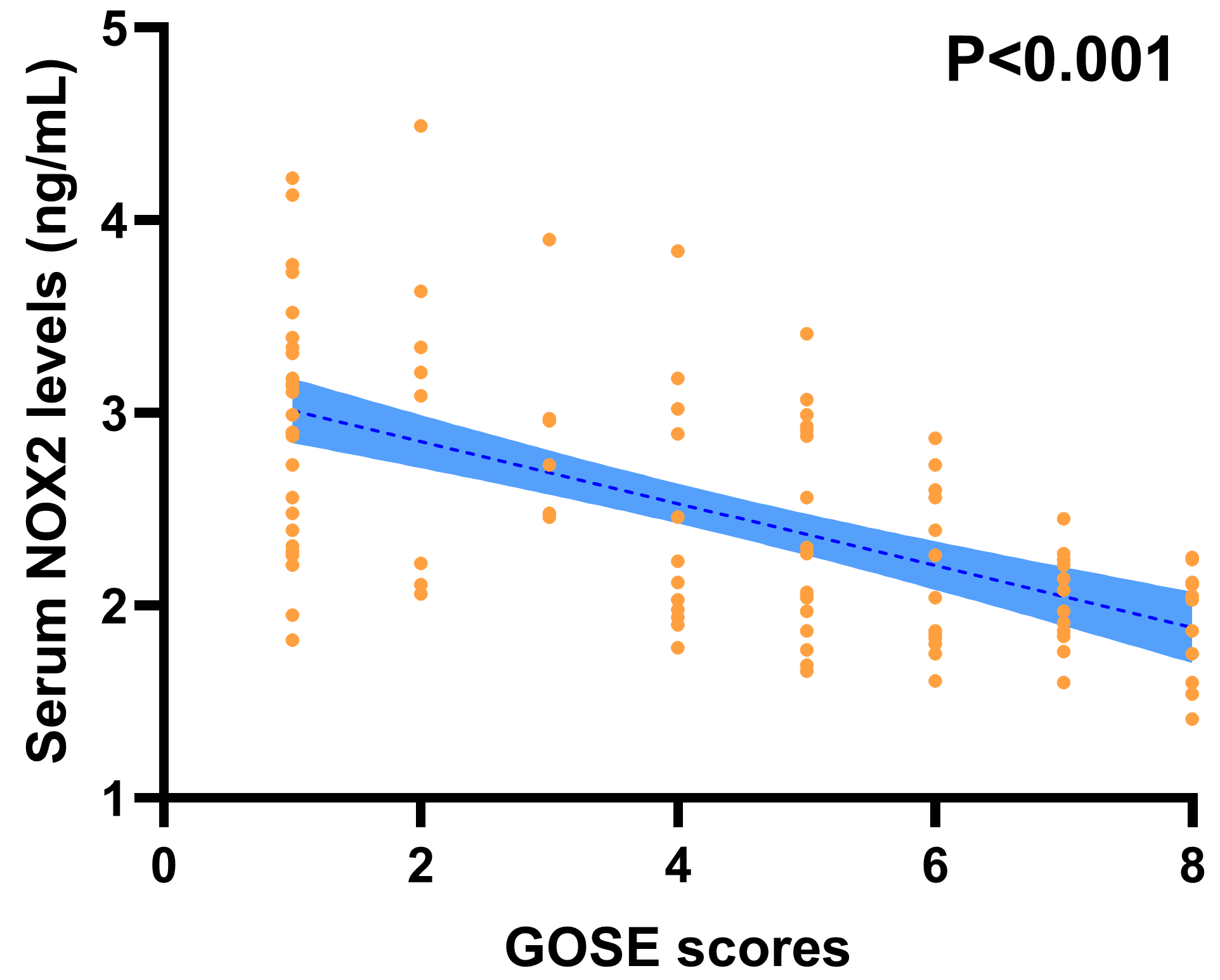

Supplement: Supplementary file 7 — Supporting fig.7 Correlogram depicting the relationship between serum NOX2 levels and 180‐day GOSE scores after sTBI. Serum NOX2 levels of patients were highly related to GOSE scores at the 180‐day mark following sTBI (P < 0.001). NOX2 indicates serum nicotinamide adenine dinucleotide phosphate oxidase 2; GOSE, Glasgow Outcome Scale Extended; stbi, severe traumatic brain injury. [file BRB3-15-e70692-s002.tif]

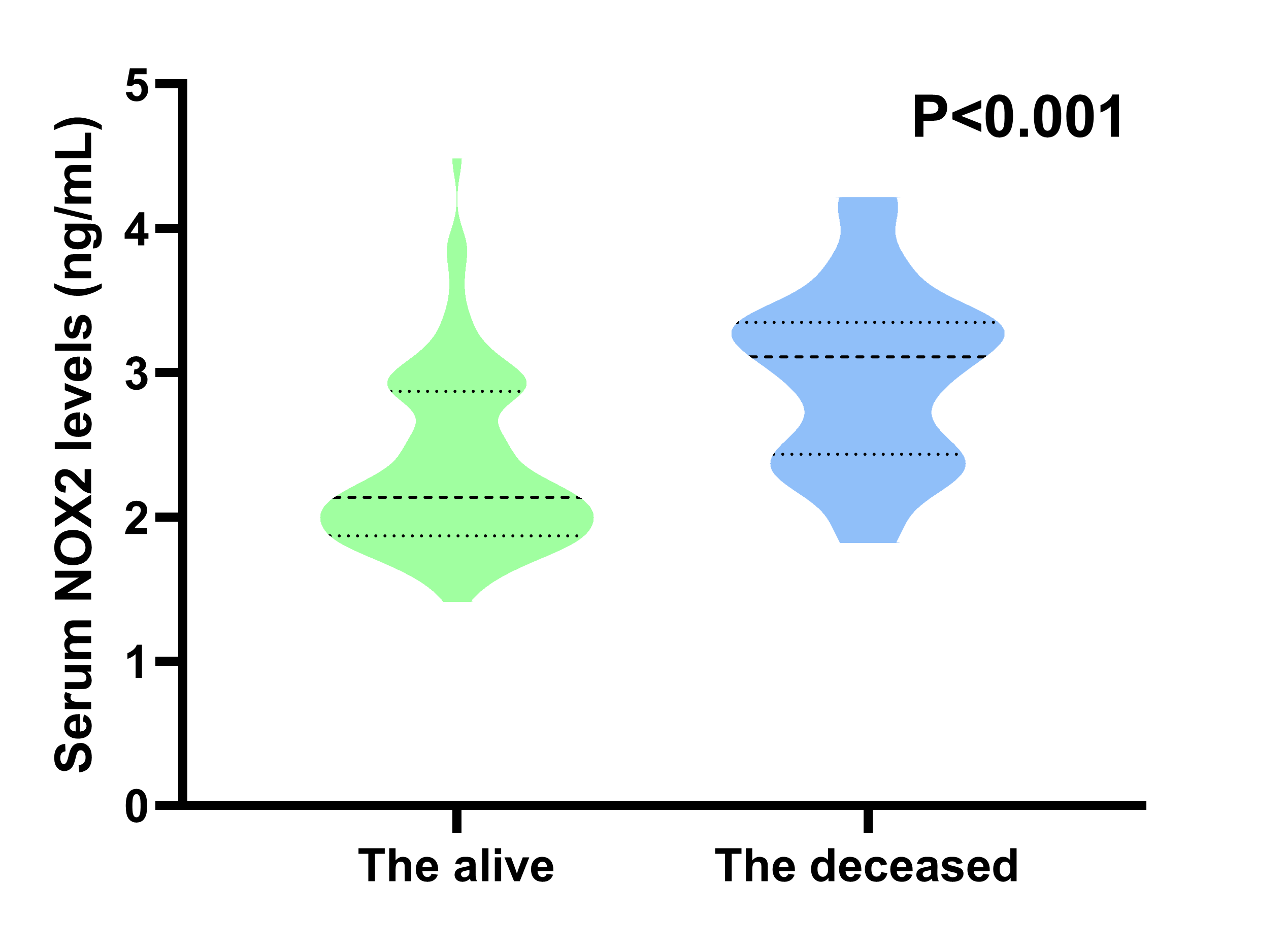

Supplement: Supplementary file 8 — Supporting fig.8 Serum NOX2 levels between the alive and the deceased at the 180‐day mark following sTBI. The boxplot showed that serum NOX2 levels of non‐survivors were substantially higher than those of survivors at 180 days post‐sTBI (P < 0.001). NOX2 signifies nicotinamide adenine dinucleotide phosphate oxidase 2; sTBI, severe traumatic brain injury. [file BRB3-15-e70692-s004.tif]

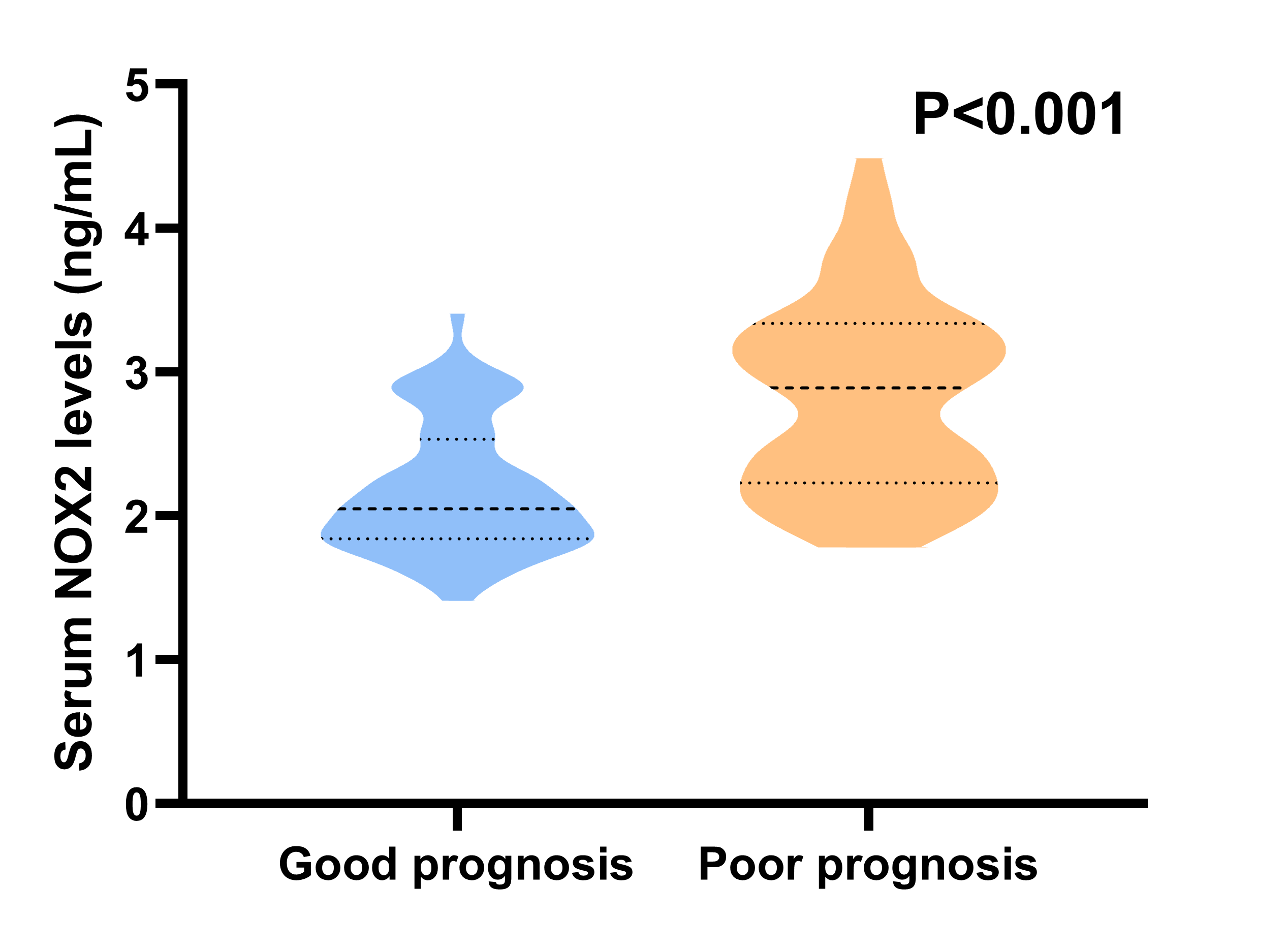

Supplement: Supplementary file 9 — Supporting fig.9 Boxplot displaying serum NOX2 levels between patients with good prognosis and those presenting with poor prognosis at 180 days after sTBI. Serum NOX2 levels were significantly higher in subjects with poor prognosis than those with good prognosis at 180 days after sTBI (P < 0.001). NOX2 stands for nicotinamide adenine dinucleotide phosphate oxidase 2; sTBI, severe traumatic brain injury. [file BRB3-15-e70692-s007.tif]
